# A bacterial-type cardiolipin synthase in *Plasmodium* spp. supports mitochondrial respiration and is important for liver stage maturation

**DOI:** 10.1371/journal.ppat.1014215

**Published:** 2026-05-11

**Authors:** Karavadra Asha Adhur, Nirdosh Nirdosh, Aakash Chandramouli, Swarnali Basu, Amit Lahiri, Siddhesh Shashikant Kamat, Satish Mishra, Saman Habib

**Affiliations:** 1 Division of Biochemistry and Structural Biology, CSIR-Central Drug Research Institute, Lucknow, India; 2 Academy of Scientific and Innovative Research (AcSIR), Ghaziabad, India; 3 Division of Molecular Microbiology and Immunology, CSIR-Central Drug Research Institute, Lucknow, India; 4 Department of Biology, Indian Institute of Science Education and Research, Pune, India; 5 Division of Pharmacology, CSIR-Central Drug Research Institute, Lucknow, India; Drexel University College of Medicine, UNITED STATES OF AMERICA

## Abstract

The mitochondrion of malaria-causing *Plasmodium* spp. supports parasite energy requirements, pyrimidine and ubiquinone biosynthesis and [Fe-S] formation. As parasites transition from the host liver to asexual and sexual blood stages, metabolic shifts of ATP generation through glycolysis or mitochondrial oxidative phosphorylation are accompanied by change in mitochondrial number, branching complexity and development of cristae. The final step of synthesis of cardiolipin (CL), a critical phospholipid for mitochondrial biogenesis and function, is catalyzed by cardiolipin synthase (Cls). *Plasmodium* spp. carry an uncharacterized, putative bacterial-type Cls distinct from Cls of mammalian hosts. We probed enzyme activity of the phospholipase D-type recombinant *Plasmodium falciparum* Cls. Antibodies generated against *Pf*Cls localized it to the mitochondrion in asexual blood stages; additional *Pf*Cls signal was observed in the cytosolic periphery in late-gametocytes, accompanied by CL staining in the parasite plasma membrane. To investigate the impact of Cls on parasite life cycle progression, we generated its knockout in the rodent parasite *P. berghei*. *Pb*Cls KO parasites had significantly impaired asexual blood-stage proliferation associated with lower abundance of CL molecular species. They showed a marked reduction in mitochondrial membrane potential and basal oxygen consumption rate. While *Pb*Cls-deficient parasites completed development within the mosquito and generated sporozoites capable of hepatocyte invasion, they exhibited a severe defect in liver-stage maturation. *Plasmodium* Cls is thus a vital component of malaria parasite development with a critical role in maintaining mitochondrial function.

## Author summary

The single mitochondrion in the liver and asexual blood stages of malaria parasites multiplying in the human host supports parasite growth and multiplication. It adopts complex branched morphology in these stages, but mitochondrial biogenesis through the parasite life cycle is not well understood. We thus sought to characterize the putative bacterial-type cardiolipin synthase (Cls) in *Plasmodium* spp. which is predicted to synthesize cardiolipin (CL), a specialized phospholipid of the inner mitochondrial membrane. Biochemical characterization and subcellular localization of Cls from the human parasite *Plasmodium falciparum* was augmented by studies on the phenotypic effects of genetic ablation of Cls in the rodent parasite *P. berghei*. The relatively slow-growing Cls knockout parasite blood stages had lower abundance of CL and phosphatidylethanolamine, as well as reduced mitochondrial membrane potential and basal respiration rate. Our results show that *Plasmodium* Cls is a phospholipase D-like enzyme whose loss results in slow replication of blood stages and a severe defect in maturation of liver-stage parasites, thus underscoring its importance for mitochondrial biogenesis and function.

## Introduction

The development and division of *Plasmodium falciparum* in human hepatocytes and erythrocytes requires extensive biogenesis of membrane compartments in the multiple daughter cells produced by schizogony. To supply the required lipids, the parasite either gets them from the host or synthesizes new lipid species de novo [1,2]. The major functional lipid classes in the malaria parasite are phospholipids, neutral lipids, sphingolipids and free cholesterol [3]. Amongst these, phospholipids represent the major lipid class in *P. falciparum* infected red blood cells (iRBC). Phospholipids also play a role in lipid signaling, formation of specialized membrane domains, anchoring of proteins to the membrane, and regulation of membrane protein structure and function [4,5]. Upon infection, the total lipid abundance in iRBCs increases 3–5 fold compared to uninfected RBCs [3], and the phospholipid content increases ~5–7 fold [6]. Since RBCs do not synthesize phospholipids, this suggests high de novo phospholipid synthesis efficiency in the parasite. There are characteristic lipid profiles for different parasite stages with lipid composition changing during differentiation and maturation of sexual stages (gametocytes) within RBCs [7]. De novo synthesis of sphingolipids and phospholipids in *Plasmodium* spp. is essential for blood stages; hence, proteins involved in biosynthesis of these lipids are potential drug targets [3,8].

**Fig 1:**
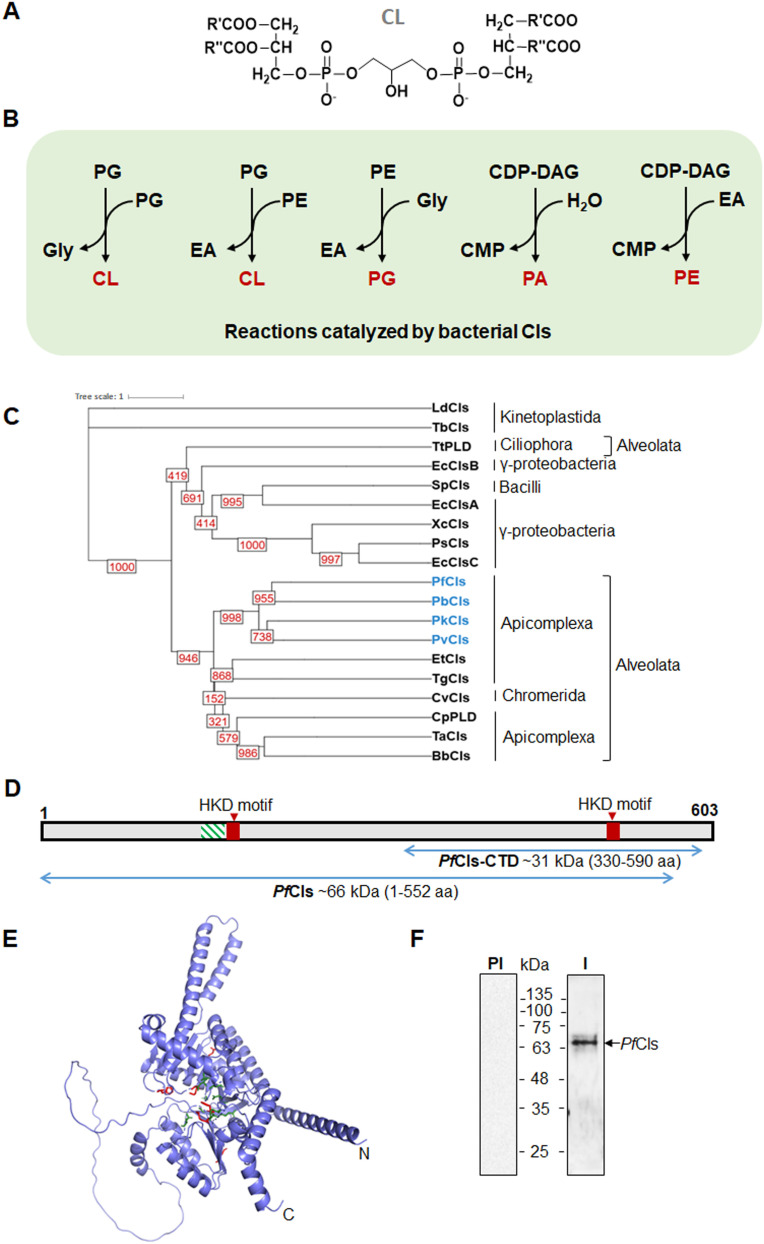
Cls activity and *Pf*Cls domain organization. **(A)** Chemical structure of cardiolipin. **(B)** Reactions catalyzed by bacterial-type cardiolipin synthases. PG or PE can transfer a phosphatidyl group to PG and form CL. Cls can also catalyze PG formation from PE and glycerol. Phospholipase D activity of Cls leads to formation of PA by hydrolyzing CDP-DAG. Cls can also use CDP-DAG with EA for PE formation, as demonstrated for *P. syringae* and *X. campestris* Cls by radio-labelling in vivo and in extracts of *E. coli* expressing the enzymes. EA, cytidine monophosphate (CMP) and glycerol (Gly) are by products in these reactions. **(C)** Phylogeny of Cls homologs from Bacteria, Alveolata and Kinetoplastida generated by PhyML 3.0. Bootstrap values of 1000 replicates are indicated at each branch. Putative Cls of *Plasmodium* spp. are in blue. Sequences used in phylogeny are provided in S1 Table. **(D)** Domain organization of the *Pf*Cls. The characteristic HKD motifs are indicated in red. The hydrophobic region suggesting presence of a putative TMD is marked with green hatched box. The *Pf*Cls stretches expressed as recombinant proteins are indicated by blue arrows. **(E)** *Pf*Cls AlphaFold model with the HKD motif residues H187, K189, D194 (HKD motif 1), H478, K480, D485 (HKD motif 2) shown as red sticks. The extended HKD motif residues R185, R188, G200, N203, G492, N495, D497, S500, E506 are in cyan. **(F)** Anti-*Pf*Cls serum recognizes a band at the expected size of full-length *Pf*Cls (~71 kDa) and a major processed form of ~68 kDa in western blot of parasite lysate from blood-stage trophozoites. I, immune serum, PI, preimmune serum.

The phospholipids phosphatidylglycerol (PG) and cardiolipin (CL) are abundant in bacterial membranes, but in eukaryotes these lipids are mostly confined to the mitochondrial membrane. Although the primary location of CL is mitochondria, it is also found in yeast and plant peroxisomes [9]. In the unicellular eukaryote *Tetrahymena thermophila,* CL is also present in the nuclear membrane and aids in nuclear expansion [10]. Among the major differences between the outer and inner mitochondrial membranes (OMM and IMM) in mammals is that the cardiolipin (CL) concentration in IMM is much higher, with CL comprising 15–20% of the total IMM phospholipid mass compared to <3% in OMM. CL is a unique anionic glycerophospholipid with a dimeric structure consisting of three glycerol groups, two phosphatidyl moieties and four acyl chains [11] (Fig 1A). It forms a hexagonal phase due to its biophysical properties and localizes to membranes with high curvature, such as in cristae in the inner mitochondrial membrane, and at septa and poles in bacteria [12,13]. In mitochondria, CL is tightly associated with respiratory complexes III and IV as well as protein transporters and plays a role in structure and function of these complexes [14,15]. CL also contributes to mitochondrial morphology, fission, fusion, cellular signalling and mitophagy [16].

CL biosynthesis is initiated through the conversion of phosphatidic acid (PA), the main precursor of phospholipid biogenesis, to cytidine di-phosphate diacyl glycerol (CDP-DAG) by the action of CDP-DAG synthase. Phosphatidyl group transfer to glycerol-3-phosphate from CDP-DAG leads to the formation of phosphatidylglycerophosphate (PGP), a reaction catalysed by PGP synthase. PGP is then dephosphorylated to PG by PGP phosphatase [17]. In most bacteria, PG receives a phosphatidyl group from another PG to form CL; on the other hand, a phosphatidyl group from CDP-DAG is transferred onto PG in most eukaryotes. These reactions are catalyzed by prokaryotic- and eukaryotic-type cardiolipin synthases (Cls), respectively. Both types of Cls belong to different enzyme classes, with distinguishing catalytic motifs. Bacterial-type Cls belong to the phospholipase D (PLD) family whereas eukaryotic-type Cls belong to the CDP-alcoholphosphatidyltransferase family [6,18]. However, there are exceptions to this Cls classification. Bioinformatics analyses indicate that *Streptomyces coelicolor* and most actinobacteria possess eukaryotic-type Cls, while eukaryotic protozoan parasites contain bacterial-type Cls [17,19,20]. *Escherichia coli* has three genes encoding Cls; Cls A utilizes two PG molecules to make CL [21], ClsB can additionally utilize PE and glycerol to form PG [22], and ClsC uses PE as phosphatidyl donor with PG for CL formation [23]. *Pseudomonas syringae* Cls not only utilizes all the known *E. coli* Cls substrates for CL formation but is also able to convert ethanolamine (EA) into phosphatidylethanolamine (PE); it also hydrolyzes CDP-DAG to yield phosphatidic acid (PA) indicating conserved PLD activity [18]. Cls can thus form a range of products based on availability of substrate (Fig 1B).

*Plasmodium* spp*.* have a single mitochondrion with changing morphology in blood, and is heavily branched prior to schizogony in the liver stages [24–26]. There is a metabolic shift of ATP generation from glycolysis in the asexual blood stages to mitochondrial oxidative phosphorylation and TCA cycle function upon transition to the sexual gametocyte stage; this is accompanied by mitochondrial enlargement, development of distinct cristae in gametocytes and increase in number to tightly clustered 6–9 mitochondria per gametocyte [25–28]. Moreover, the prevalence of components of the respiratory chain complex and associated metabolic pathways is enhanced many-fold in gametocytes [25]. In comparison to other organisms, CL species in *P. falciparum* blood stages comprise of unusually long-chained, even-numbered side-chain fatty acids with structural diversity due to the variable number of total double bonds [29]. Among the enzymes of the conventional CL biosynthesis pathway, a PGP phosphatase is not identifiable in the *P. falciparum* genome [6]. The single Cls encoded by apicomplexan parasites, including *P. falciparum,* belongs to the PLD family with homology to bacterial-type Cls [17,20] and thus differs from Cls of the human host. We therefore probed the putative *Pf*Cls biochemically to identify potential lipid substrates, determined its localization in blood stages of parasite development, and examined the consequences of genetic ablation of *Plasmodium* Cls on parasite development across its life cycle.

## Results

### A bacterial type Cls is expressed in *P. falciparum*

Putative Cls from apicomplexan parasites form a clade distinct from the bacterial and kinetoplastid Cls in phylogenetic analysis of selected PLD-type Cls, with *Plasmodium* spp. forming a tight cluster (Fig 1C). The putative bacterial-type Cls of *P. falciparum* (PlasmoDB ID: PF3D7_0609400) has 17% and 23% sequence identity with *Streptococcus pneumoniae* Cls and *E. coli* ClsB, respectively (S1A Fig). *Pf*Cls contains two highly conserved H(X)K(X)_4_D motifs found in the active sites of phospholipase D (PLD), nucleases, and poxvirus envelope proteins [30]. The minimal H(X)K(X)_4_D motifs in *Pf*Cls are part of two signature PLD domains, R(X)HRK(X)_4_D(X)_5–6_G(X)_2_N and H(X)K(X)_4_D(X)_6_G(X)_2_N(X)D(X)_2_S(X)_4–5_E, that are common to most bacterial Cls (Figs 1D and S1A) [17,31]. Cls homologs in kinetoplastids such as *Trypanosoma brucei* and apicomplexan parasites including *Cryptosporidium parvum* have conserved PLD domains (S1A Fig). The AlphaFold [32] generated model of *Pf*Cls indicates conservation of folds with an unstructured loop from residues 222–279 and low model confidence score at the N- and C-termini of the protein (S1B Fig). The active site pocket with the HKD motifs of *Pf*Cls is similar to known structures of the PLD family proteins (Fig 1E). A mitochondrial targeting sequence (MTS) is not identified at the N-terminus (MitoProt II score: 0.34).

In order to check expression of the *Cls* gene in *P. falciparum*, we generated antibodies against the C-terminal domain (330–590 aa) of the protein in rabbit (Figs 1D and S1C). Specificity of the generated Abs was confirmed by probing lysates from *E. coli* cells expressing *Pf*Cls-CTD and those transformed with the empty vector (S1D Fig). The Abs were used to detect *Pf*Cls in western blots of the parasite lysate from asexual blood stages. A major band of ~68 kDa was detected by the anti-*Pf*Cls serum together with an upper fainter band, the latter corresponding to the expected size of full-length *Pf*Cls (~71 kDa) (Fig 1F). The lower band is likely to be a processed form of the protein although another post-translational modification is not ruled out. The single *cls* gene identified in *P. falciparum* lacks an intron, thus minimizing the likelihood of an isoform. No signal was seen in control western blot with rabbit pre-immune serum. *Pf*Cls is thus expressed in the parasite blood stages.

Cls is an integral membrane protein in bacteria and eukaryotic cells with distinct transmembrane domains identified in *E. coli* ClsA [23], *Saccharomyces cerevisiae* [33] and *Trypanosoma brucei* Cls [17]. *E. coli* ClsB and C lack predicted transmembrane helices but seem to be associated with the bacterial membrane [23]. Although a hydrophobic stretch between residues 163–185 is detected in *Pf*Cls, a clear prediction is not made for presence of a transmembrane domain (S2A Fig). In immunofluorescence assays (IFA) of *E. coli* cells expressing recombinant *Pf*Cls, the protein localized at the cell periphery suggesting that it is associated with the bacterial membrane (S2B Fig). Differential extraction of *Pf*Cls from *P. falciparum* trophozoites showed partitioning of the protein in the Tris-soluble, membrane peripheral (carbonate soluble), membrane intrinsic and insoluble (Triton X-100 soluble and insoluble) fractions compared to the control protein β-actin which expectedly partitioned in the Tris-soluble and carbonate-soluble fractions alone (S2C Fig). *Pf*Cls partitioning across fractions with differential solubility suggests that it primarily exists as a membrane-associated protein with an additional presence outside the cellular membrane component. In vivo DSP-crosslinking followed by DTT-mediated release showed *Pf*Cls as part of a large complex (S2D Fig), suggesting that it forms homo- and/or hetero-meric membrane-associated complexes.

### *Pf*Cls and cardiolipin have stage-dependent distribution in the mitochondria and cellular periphery in *P. falciparum* asexual and sexual blood stages

**Fig 2:**
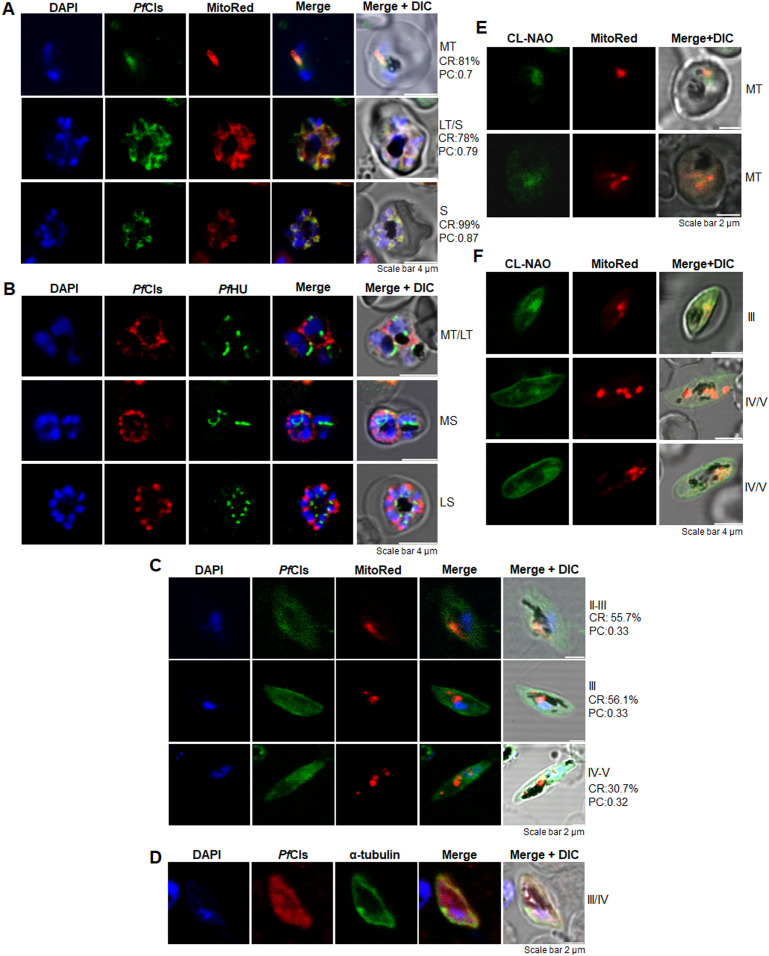
Subcellular localization of *Pf*Cls and distribution of CL through asexual and sexual blood stages. **(A, B)** Immunofluorescence confocal microscopy for localization of *Pf*Cls in asexual blood stages using anti-*Pf*Cls antisera. MitoTracker Red is the mitochondrial marker dye in (A). *Pf*HU is the apicoplast marker in (B), DAPI is the nuclear dye. DIC, differential interference contrast; MT, mid-trophozoites; LT, late-trophozoites; S, schizonts; MS, Mid-schizonts; LS, late-schizonts. The co-localization rate (CR, %) and Pearson’s coefficient (PC) between *Pf*Cls and MitoTracker Red is given in (A). The scale bar is 4 µm. The images are representative of >20 images scanned from three sets each for (A) and (B). **(C)** Localization of *Pf*Cls at different gametocyte stages (II-V) with MitoTracker Red as the mitochondrial marker dye. The images are representative of >50 scans from two experiments. **(D)** Partial overlap of *Pf*Cls with α-tubulin signal below the parasite plasma membrane in gametocytes. The images are representative of >50 scans from two experiments. **(E)** Blood stage trophozoites stained with NAO and MitoTracker Red. The scale bar is 2 µm. The images are representative of >30 images. **(F)** Gametocytes (stage III-V) stained with NAO and MitoTracker Red. The scale bar is 4 µm. The images are representative of >90 images scanned for gametocyte stages.

Subcellular partitioning of *Pf*Cls to the trophozoite organellar fraction was indicated by the thermolysin protection assay (S2E Fig) where *Pf*Cls and the apicoplast-resident *Pf*HU were protected from digestion by the protease in digitonin-treated cells, while the cytosolic control protein tubulin was degraded. All three proteins were intact when thermolysin was inhibited by EDTA. The partial thermolysin protection of *Pf*Cls compared to *Pf*HU, a resident of the four membrane-bound apicoplast, suggested that *Pf*Cls localized to another organelle which was differentially susceptible to digitonin permeabilization. The localization of *Pf*Cls across asexual blood stages and gametocytes was determined by immunofluorescence assays (IFA) using anti-*Pf*Cls Ab. *Pf*Cls signal overlapped with the mitochondrial marker dye (Pearson’s coefficient = 0.756 ± 0.11) in the mid- to late-trophozoite and schizont stages, localizing within the elongated/branched mitochondrion in trophozoites (Fig 2A) and partitioning to individual merozoites in late schizonts (Fig 2B). No overlap of the *Pf*Cls signal was seen with the apicoplast marker *Pf*HU (Fig 2B). *Pf*Cls thus localized to the mitochondrion in asexual blood stages. In gametocytes (stage III-V), the *Pf*Cls signal overlapped with the mitochondrial marker, but prominent signal was also seen in the cytosol and towards the cell periphery (Fig 2C and 2D). *Pf*Cls signal partially overlapped with tubulin below the parasite membrane (Fig 2D). These results suggested that *Pf*Cls is completely mitochondrial in asexual blood stages but has additional extra-mitochondrial localization in late-gametocytes, where it also accumulates at the cytosolic periphery. The partial thermolysin protection of *Pf*Cls compared to apicoplast *Pf*HU in blood stages could be a result of differential permeabilization of the two-membrane bound mitochondrion compared to the four membrane-bound apicoplast by digitonin.

The unusual dual localization of *Pf*Cls in gametocytes prompted us to check the cellular distribution of CL in blood stage parasites. 10-N-nonyl acridine orange (NAO), a high affinity probe for CL detection which has low affinity for other anionic phospholipids and does not bind zwitterionic phospholipids, was used with MitoTracker Red. NAO emits green fluorescence at 525 nm at low levels of CL [34–36]. NAO signal overlapped with MitoTracker Red in trophozoites (Fig 2E). However, in gametocytes, NAO fluorescence co-localized with the mitochondrial dye and also appeared at the parasite plasma membrane (PPM) (Fig 2F). A more prominent membrane signal for NAO was seen at late (stage IV/V) gametocytes, suggesting the presence of CL in the PPM in late gametocyte stages. NAO signal in the PPM of gametocytes, but not of trophozoites, indicated that weak NAO binding to anionic phospholipids other than CL contributed only minimally to the observed fluorescence; levels of anionic phospholipids such as PG and PS actually decrease in late gametocytes compared to trophozoites [3,7].

### *Pf*Cls has phospholipase D activity and can enhance CL levels in *E. coli*

**Fig 3:**
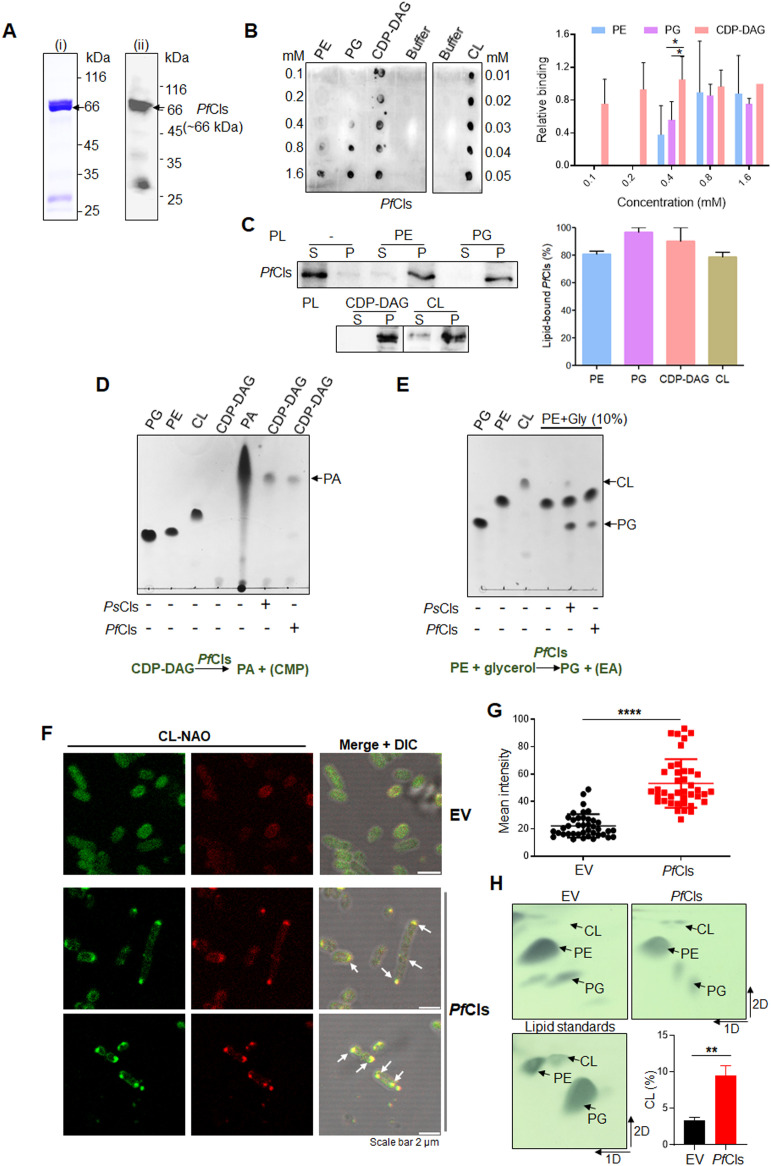
Biochemical characterization of recombinant *Pf*Cls. **(A)** Coomassie-stained SDS-PAGE of purified recombinant *Pf*Cls (i), and the corresponding western blot probed with anti-*Pf*Cls serum (ii). **(B)** Protein-lipid overlay assay to check binding of recombinant *Pf*Cls with different concentrations of phospholipids: PG, PE, CDP-DAG and CL. Buffer alone served as negative control; the buffer column is shown in both panels of the blot to show similar exposure. The blot was probed with anti-6XHis Ab. The corresponding graph shows quantification of concentration-dependent binding from three repeat protein-lipid overlay experiments. Mean ± SD are plotted. * indicate P = 0.045 and 0.029 for comparison of binding to CDP-DAG versus PG and PE, respectively. **(C)** Co-sedimentation of *Pf*Cls with different phospholipid (PL) liposomes. The supernatant (S) and pellet (P) were probed with anti-6XHis Ab for detection of *Pf*Cls. The graph shows quantification of lipid-bound *Pf*Cls (%). Mean ± SD from three repeat experiments is plotted. **(D)** Conversion of CDP-DAG to PA indicates phospholipase D activity of *Pf*Cls. Lanes 1-5 of the TLC are lipid standards. *P. syringae* Cls was used as positive control. CMP, cytidine monophosphate. **(E)** *Pf*Cls catalyzes formation of PG from PE and glycerol. *Ps*Cls was the positive control enzyme. Representative images from three repeat experiments are shown for (D) and (E). **(F)** CL synthesis by *Pf*Cls in *E. coli*. Fluorescence confocal microscopy of late exponential phase bacteria transformed with pET23a(+)-*Pf*Cls (*Pf*Cls) or the empty vector pET23a(+) (EV) under identical scan settings. CL was detected by NAO staining. CL-rich regions in *Pf*Cls expressing cells are shown by solid arrows. Scale bar is 2 µm. **(G)** Mean fluorescence intensity calculated for CL-rich regions-- cell septum and cell poles of bacteria in (F). Mean ± SD are plotted for 40 cells each. P value (P < 0.0001) was calculated by the Mann-Whitney non-parametric two-tailed test. **(H)** 2D-TLC for detection of CL levels in *E. coli* expressing *Pf*Cls or transformed with the empty vector. CL, PE and PG were used as phospholipid standards. Dimensions of the mobile phase are indicated by solid arrows. CL levels are plotted as % of total phospholipids from three experiments; P < 0.01.

In order to investigate the biochemical activity of *Pf*Cls, we first expressed full-length *Pf*Cls (1–603 aa) as 6X-His tagged protein in *E. coli*. However, the protein was highly unstable and could not be purified. We then expressed a slightly shorter version (1–552 aa) as a 6XHis-fusion protein (S3A Fig), removing the C-terminal end which is not conserved in Cls homologs from other genera (S1A Fig). Although the levels of *Pf*Cls expression in *E. coli* were low, it purified as two closely migrating bands at the expected size (~66 kDa) with a minor degraded product of >25 kDa (Fig 3A). The two bands at ~66 kDa were recognized by ant-6XHis Abs (S3A Fig) indicating that the lower band was an N-terminal degradation product/processed form of the full-length protein. The two bands could not be cleanly separated and the affinity-purified *Pf*Cls comprising both forms was used in activity experiments. Upon size exclusion chromatography, *Pf*Cls eluted at the expected size of the globular protein, with peak 1 containing the main bands of ~66 kDa and peak 2 with additional degradation products (S3B Fig). Non-aggregation of *Pf*Cls was indicated by the absence protein elution in the void volume (before 8 ml) (S3B Fig). Peptide mass spectrometry of the peak 1 sample confirmed the correct identity of the protein (S1 Table). Secondary structure of purified *Pf*Cls was confirmed by CD spectroscopy; CD spectrum of *Pf*Cls showed a dip at 222 nm indicating the expected presence of α-helices (S3C Fig).

Recombinant *Pf*Cls was used to assess binding with known phospholipid substrates PE, PG, CDP-DAG and the product CL by the protein-lipid overlay assay (Fig 3B) and liposome co-sedimentation assay (Fig 3C). In the former assay, *Pf*Cls interacted with all lipids in a lipid concentration-dependent manner with maximal retention of *Pf*Cls on the membrane seen with CL and CDP-DAG (Fig 3B). No signals were seen with the buffer alone or with the negative control probe protein *Pf*Exo [37] (S3D Fig). Of the three lipid substrates, *Pf*Cls showed significantly higher binding to CDP-DAG (Fig 3B). To further confirm *Pf*Cls binding to lipid layers, *Pf*Cls interaction with liposomes comprising of different phospholipids was checked by co-sedimentation. In the absence of liposomes, most *Pf*Cls was in the soluble fraction with low amounts in the pellet (Fig 3C). *Pf*Cls co-sedimented with the liposome pellet for all lipids tested, with comparable (78–96%) partitioning in the pellet fraction (Fig 3C). *Pf*Cls can thus bind potential lipid substrates as well as its product, cardiolipin.

The biochemical activity of *Pf*Cls was tested using different known phospholipid substrates for bacterial Cls in standard assay conditions followed by thin-layer chromatography (TLC) detection [18]. Although CL formation was seen with the positive control *P. syringae* Cls (*Ps*Cls) (S3E Fig), we could not detect CL formation even with five times higher concentration of *Pf*Cls in the reactions with PG alone or PG and PE as substrates (S3F Fig). *Pf*Cls, however, was able to hydrolyze CDP-DAG to PA, indicating that it had PLD activity as observed in *Ps*Cls (Fig 3D) [18]. *Pf*Cls could additionally convert PE and glycerol to PG by transphosphatidylation (Fig 3E), a catalytic property also found in *E. coli* ClsB [22]. PE formation from CDP-DAG and EA was not detected by either purified *Ps*Cls or *Pf*Cls (S3G Fig). Our failure to detect CL generation by recombinant *Pf*Cls from PG and PE in vitro might be attributable to weak activity of the enzyme and/or high CL detection limit in TLC staining.

To determine whether *Pf*Cls supports CL synthesis in vivo, we compared CL levels in *E. coli* cells expressing recombinant *Pf*Cls or transformed with the expression vector backbone alone, and harvested in the late exponential phase of bacterial growth. CL was detected by NAO staining. While NAO emits green fluorescence, high CL levels cause aggregation of the dye resulting in Stokes shift of fluorescence from 525 nm to red fluorescence at 640 nm [34]. Bacterial membrane phospholipids comprise of 70–80% PE, 20–25% PG and ~5% CL which show heterogeneous distribution in the plasma membrane. PE and PG localize to cylindrical regions of rod-shaped bacteria such as *E. coli* [38–40], with CL tending to localize to regions with high negative curvature which are poles and septum in bacteria [12,41]. In comparison to vector control, which showed NAO signal mostly at green emission, *Pf*Cls-expressing cells had higher NAO fluorescence at both green and red wavelengths indicative of enhanced CL levels (Fig 3F). NAO fluorescence in *Pf*Cls-expressing cells was localized to the plasma membrane with intense signals at the bacterial poles and septum (Fig 3F). Red NAO fluorescence at the bacterial poles was significantly higher in *Pf*Cls-expressing cells (Fig 3G). Quantification of bacterial CL after two dimensional-TLC also showed that *Pf*Cls-expressing cells had significantly higher CL content (9.5 ± 1.9% of total lipid) compared to bacteria transformed with empty vector (3.3 ± 0.6% of total lipid), P < 0.01 (Fig 3H). Thus, the presence of *Pf*Cls in *E. coli* enhances CL levels in the bacterial cell membrane, suggesting its direct role in CL synthesis. However, the possibility that increase in CL is the result of an indirect effect, such as stimulation of an *Ec*Cls, cannot be ruled out.

### Generation of *PbCls* knockout and complemented parasite lines

There is 63% sequence identity between *P. falciparum* and *P. berghei* Cls, with conservation of the PLD domain and HKD motifs (S1A Fig). We thus investigated the role of Cls in the *Plasmodium* life cycle, by generating a knockout parasite line using a double-crossover homologous recombination strategy (S4A Fig). After transfection, recombinant parasites were selected with pyrimethamine, and integration of the GFP selection cassette was confirmed by fluorescence microscopy (S4B Fig). Site-specific disruption of the *PbCls* locus was validated by diagnostic PCR (S4C Fig). Clonal parasite lines were obtained by limiting dilution from two independent transfections and used for subsequent analyses. To assess the specificity of the knockout phenotype, we generated a complemented line by reintroducing the *Pb*Cls coding sequence under its endogenous regulatory elements at the disrupted locus (S4D Fig). Restoration of the wild-type locus was confirmed by diagnostic PCR (S4E Fig). The complemented parasites (*Pb*Cls-comp) displayed no detectable defects and phenocopied WT-GFP parasites throughout the parasite life cycle. *Pb*Cls in wild type parasites was cross-recognized by the anti-*Pf*Cls Ab in western blot (S5A Fig). Fluorescence microscopy revealed strong Cls signals in both blood-stage and late liver-stage parasites. In contrast, no signal was observed in *Pb*Cls knockout parasites (S5B and S5C Fig).

### *Pb*Cls is required for efficient blood-stage propagation and maintenance of mitochondrial respiration

**Fig 4:**
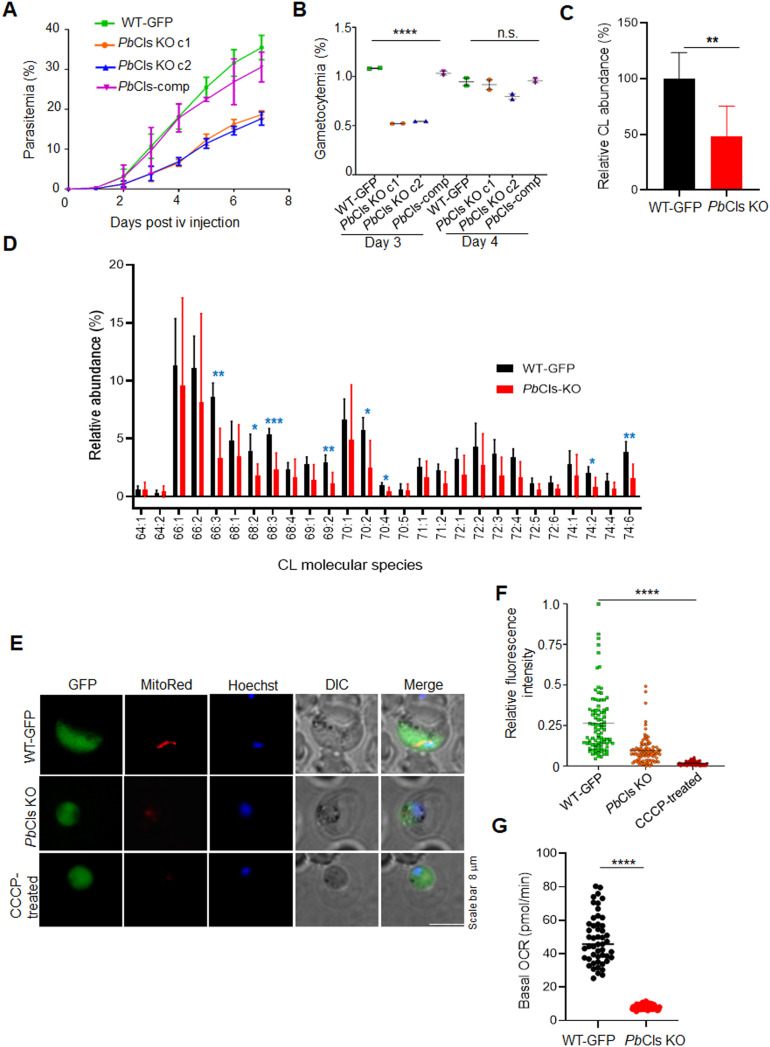
*Pb*Cls is required for asexual blood-stage growth and mitochondrial function. **(A)** Asexual growth of *Pb*Cls KO parasites is significantly reduced compared to WT-GFP and *Pb*Cls-complemented lines. Parasitemia was monitored daily following infection; KO parasites exhibited a marked proliferation defect (day 1, P = 0.0308; day 2, P = 0.4687; day 3, P = 0.0978; day 4, P = 0.0009; day 5, P = 0.0001; day 6, P = 0.0009; day 7, P = 0.0002). **(B)** Gametocytemia in *Pb*Cls KO parasites is significantly decreased on day 3 post-infection compared to WT-GFP (P < 0.0001), but recovers by day 4 (P = 0.1471). **(C)** Relative total CL levels in *Pb*Cls KO compared to WT-GFP parasites. P-value was determined by unpaired Student’s t-test (P = 0.0054). **(D)** Relative abundance of CL molecular species in WT-GFP and *Pb*Cls KO parasites. Mean ± SD of six independent biological replicates are plotted. Multiple t-test with 5% Benjamini-Krieger-Yekutieli FDR correction was applied; adjusted P-values are depicted. **(E and F)** Mitochondrial membrane potential was assessed using MitoTracker Red FM staining. *Pb*Cls KO parasites showed a significant reduction in relative fluorescence intensity compared to WT-GFP (P < 0.0001). Error bars represent mean ± SEM; data are representative of three independent experiments. Statistical comparisons were performed using one-way ANOVA. **(G)** Basal OCR of WT-GFP and *Pb*Cls KO parasites from S6F Fig showing mean ± SD of data points for basal respiration (between 1-20 min). P-value was determined by unpaired Student’s t-test (P < 0.0001).

To investigate the role of *Pb*Cls during asexual blood-stage development, we intravenously injected Swiss albino mice (n = 5 per group) with 0.5% parasitemia (200 μl) of either WT-GFP or *Pb*Cls KO parasites. Parasite proliferation was monitored daily via Giemsa-stained thin blood smears. Compared to WT-GFP parasites, *Pb*Cls KO parasites exhibited significantly reduced blood-stage growth (Fig 4A). This impaired replication was also reflected in reduced gametocytemia; however, by day 4 post-infection, gametocyte levels in the KO group approached those of WT-GFP parasites (Fig 4B). We confirmed that the observed phenotype is not due to outgrowth of a minor WT population by genotypic validation of parasites recovered at the end of blood stage development. PCR analysis confirmed the KO locus with no detectable WT contamination (S5D Fig). To determine the effect of Cls depletion on CL levels, we analyzed CL molecular species by targeted lipidomic analysis. There was a significant reduction in total CL in *Pb*Cls KO parasites compared to WT (Fig 4C). A number of CL molecular species were reduced in the *Pb*Cls KO (Fig 4D). Among the CL species detected, there was no preferential effect on species with short/long chain length or low/high saturation. We also analyzed levels of other phospholipids—PE, PG, PA, PC and PS which have roles in mitochondrial dynamics and function. While there was no significant change in abundance of PG, PA and PC in the *Pb*Cls KO, PE and PS abundance was significantly reduced (S6A–S6I Fig); the majority of detected PE and PS molecular species had lower abundance in the *Pb*Cls KO (S6G and S6I Fig).

To determine whether the impaired blood-stage growth was associated with mitochondrial dysfunction, we detected relative mitochondrial polarization using the cationic dye MitoTracker Deep Red FM. WT-GFP parasites treated with the protonophore carbonyl cyanide m-chlorophenyl hydrazone (CCCP), which collapses mitochondrial membrane potential (ΔΨm), showed near-complete loss of MitoTracker fluorescence and served as a depolarization control (Fig 4E and 4F). *Pb*Cls KO parasites displayed significantly reduced mitochondrial fluorescence compared to WT-GFP, though the signal remained higher than in CCCP-treated parasites. In order to determine whether loss of *Pb*Cls causes a defect in the mitochondrial electron transport chain (mETC) thus affecting respiration, we measured the oxygen consumption rate (OCR) using malate as substrate. Comparison of OCR of WT and *Pb*Cls KO trophozoites assayed in an XFe24 flux analyzer showed significant reduction (P < 0.0001) of basal OCR in *Pb*Cls KO parasites (Figs S6J and 4G). The partial loss of MitoTracker fluorescence as well as reduction in basal OCR indicate that mitochondrial respiration is compromised in the *Pb*Cls KO.

### Parasite development in the mosquito vector is not affected in the *Pb*Cls KO line

**Fig 5:**
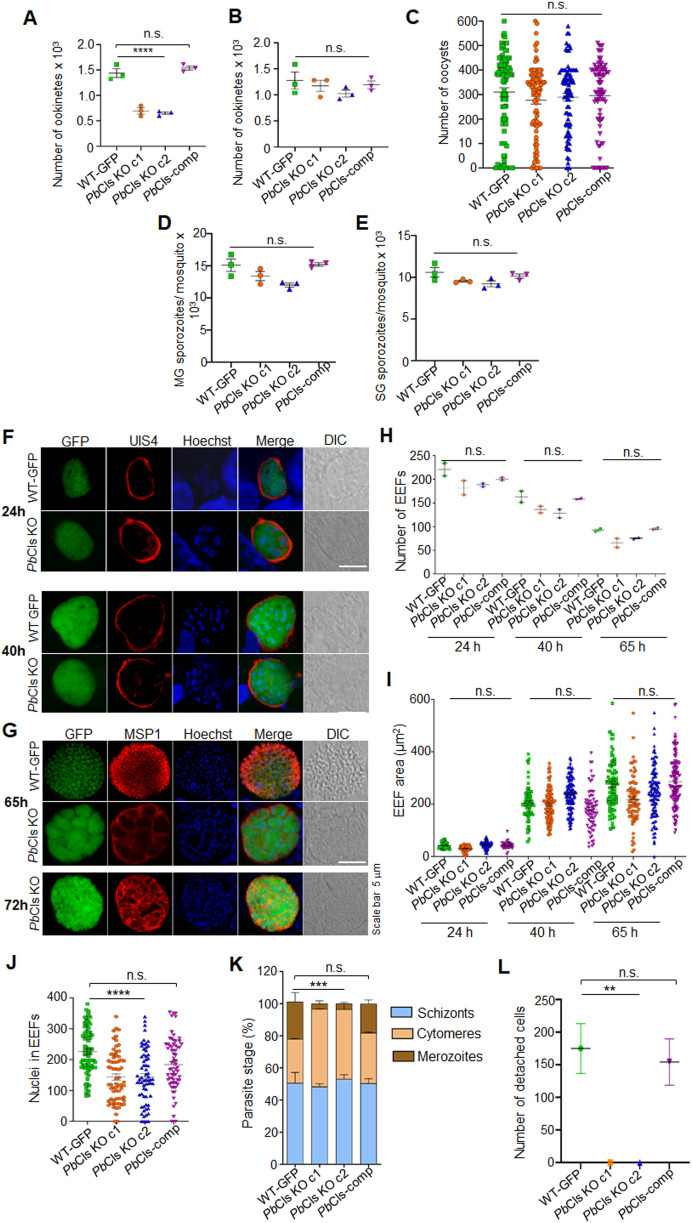
*Pb*Cls is critical for late liver-stage maturation but dispensable during early exo-erythrocytic development and mosquito stages. **(A)** Significant reduction (P = 0.0002) in ookinete numbers in *Pb*Cls KO when mosquitoes fed on mice with low gametocytemia at day 3 post infection. **(B)** No significant difference in ookinete numbers (P = 0.4762) between WT-GFP, *Pb*Cls KO, and *Pb*Cls-comp parasites when mosquitoes fed on mice at day 4 post infection. **(C)** Oocyst quantification showed no significant differences among WT-GFP, *Pb*Cls KO, and *Pb*Cls-comp parasites (P = 0.5272). **(D)** Midgut sporozoite numbers were comparable between parasite lines (P = 0.0591). **(E)** Salivary gland sporozoite quantification revealed no significant differences between WT-GFP, *Pb*Cls KO, and *Pb*Cls-comp parasites (P = 0.1035). Data represent mean ± SEM from three independent experiments. **(F and G)** HepG2 cells were infected with WT-GFP or *Pb*Cls KO sporozoites and fixed at 24, 40, and 65 hpi and immunostained with anti-UIS4 or anti-MSP1 antibodies; nuclei were stained with Hoechst 33342. **(H and I)** Quantification of EEF number and size revealed no significant difference between WT-GFP and *Pb*Cls KO parasites (EEF number: 24 hpi, P = 0.1901; 40 hpi, P = 0.1557; 65 hpi, P = 0.0959; EEF area: 24 hpi, P = 0.0276; 40 hpi, P = 0.0163; 60 hpi, P = 0.0133). **(J)** Schizont maturation was severely impaired in *Pb*Cls KO parasites at 65 hpi, as evidenced by significantly reduced nuclear counts compared to WT-GFP (P < 0.0001). **(K)** Counting of EEF stages demonstrated an arrest at the cytomere stage in KO parasites, with a significant reduction in mature merozoites (P = 0.0003). Data represent mean ± SEM from three independent biological replicates. Statistical significance was determined using one-way ANOVA. **(L)** Detached cell formation is significantly reduced in *Pb*Cls KO parasites compared to WT-GFP (P = 0.0018).

To assess the role of *Pb*Cls during mosquito stage development, we analyzed the phenotype of *Pb*Cls KO parasites. Outgrowth of a minor WT population was ruled out by PCR validation of parasites recovered at the end of mosquito-stages (S5D Fig). In initial experiments, we observed a marked reduction in the number of midgut ookinetes in mosquitoes fed on KO-infected mice (Figs 5A and S7A). However, this was accompanied by reduced gametocytemia at day 3 post-infection relative to WT-GFP controls, suggesting that the observed defects may result from delayed parasite growth rather than a direct role of *Pb*Cls in mosquito-stage development. To account for this, we repeated mosquito infections using mice with matched gametocytemia at day 4 post-infection. Under these conditions, ookinete numbers, oocyst formation and midgut sporozoite development were comparable between KO and WT-GFP parasites (Figs 5B–5D, S7B and S7C). Furthermore, salivary gland sporozoite loads at day 18 post-feeding did not differ significantly between the KO and WT-GFP parasites (Figs 5E and S7D). These data indicate that *Pb*Cls is not required for parasite transmission through the mosquito vector.

### *Pb*Cls is critical for mitochondrial biogenesis and maturation of hepatic merozoites

To determine the role of *Pb*Cls during liver-stage development, we first examined the infectivity of *Pb*Cls KO sporozoites in vivo. C57BL/6 mice were intravenously injected with 5,000 sporozoites, and parasitemia was monitored by Giemsa-stained blood smears. While all mice eventually developed blood-stage infections, the prepatent period was significantly delayed in mice infected with *Pb*Cls KO sporozoites. The delay ranged from 2.6 to 4 days compared to WT-GFP and *Pb*Cls-complemented control parasites (Table 1), suggesting a defect in liver-stage development or transition to blood stage.

**Table 1:** Infectivity of *Pb*Cls KO sporozoites in C57BL/6 mice. *Pb*Cls KO parasites exhibited a significant delay in the pre-patent period compared to controls (Kruskal-Wallis test, P = 0.0313).

Experiment	Parasites	Number of sporozoites injected	Mice positive/mice injected	Pre-patent period (days)
1	WT-GFP	5,000	5/5	3.0
	*Pb*Cls KO	5,000	5/5	5.6
	*Pb*Cls KO	5,000	5/5	6.4
	*Pb*Cls comp	5,000	5/5	3.2
2	WT-GFP	5,000	5/5	3.0
	*Pb*Cls KO	5,000	5/5	6.4
	*Pb*Cls KO	5,000	5/5	5.6
	*Pb*Cls comp	5,000	5/5	3.0
3	WT-GFP	5,000	5/5	3.2
	*Pb*Cls KO	5,000	5/5	7.0
	*Pb*Cls KO	5,000	5/5	6.4
	*Pb*Cls comp	5,000	5/5	3.0

**Fig 6:**
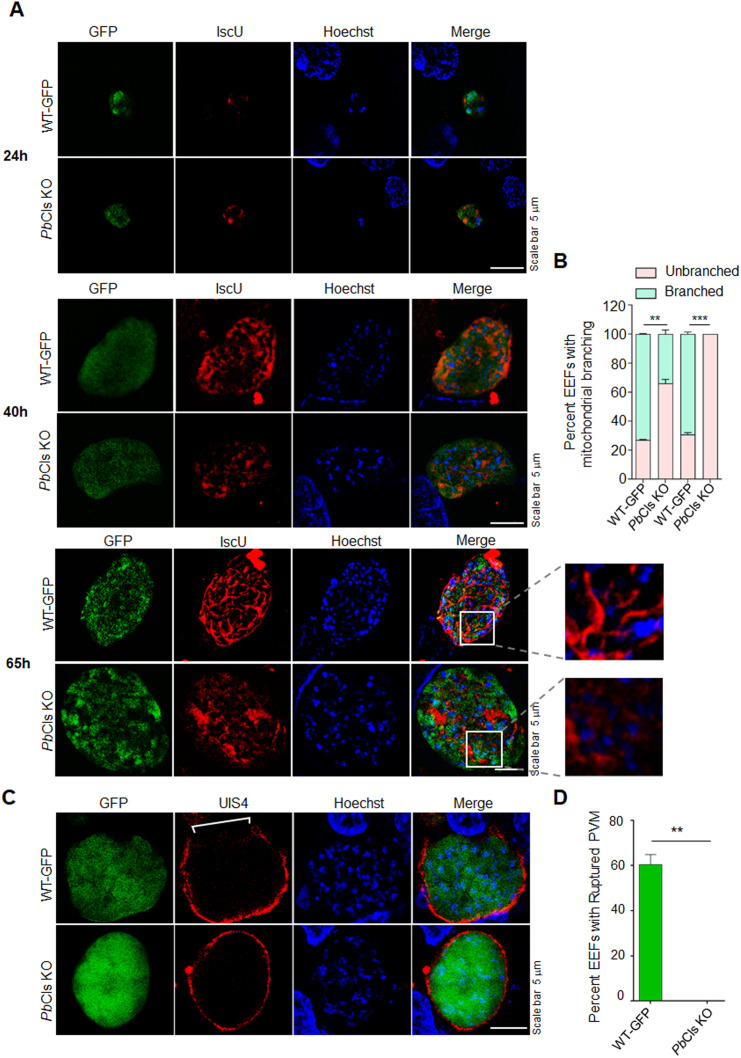
Mitochondrial branching and PVM rupture defects in *Pb*Cls KO parasites. **(A)** Immunofluorescence analysis using an anti-IscU antibody revealed impaired mitochondrial branching in *Pb*Cls KO parasites compared to WT-GFP at 40 and 65 hpi. **(B)** Quantification of mitochondrial branching in EEFs confirmed a significant reduction in *Pb*Cls KO parasites compared to WT-GFP. Mitochondria exhibiting ≥5 at 40 hpi or ≥10 branched tubular networks at 65 hpi were classified as normal, while those below these numbers were considered unbranched. Statistical analysis demonstrated a significant decrease in mitochondrial branching in *Pb*Cls KO parasites (P = 0.0052 at 40 hpi; P = 0.0003 at 65 hpi). A total of 100 EEFs at 40 hpi and 50 EEFs at 65 hpi were analyzed per group, from two independent biological replicates. **(C)** HepG2 cells infected with WT-GFP or *Pb*Cls KO parasites were fixed at 65 hpi and immunostained with anti-UIS4 antibody to label the PVM. Nuclei were stained with Hoechst. PVM rupture in WT-GFP is indicated by a white line. **(D)** Quantification of liver-stage parasites shows a significantly higher proportion of EEFs with intact PVMs in *Pb*Cls KO parasites relative to WT-GFP (P < 0.005). A total of 120 EEFs were analysed for each group from two independent biological replicates.

To investigate the cause of the observed delay in the prepatent period in *Pb*Cls KO parasites, we first quantified hepatocyte invasion by sporozoites using differential staining. No significant difference was observed between *Pb*Cls KO and WT-GFP parasites, indicating normal invasion efficiency (S8 Fig). We next examined exo-erythrocytic form (EEF) development in vitro. Cultures were fixed at 24, 40, 65, and 72 hours post-infection (hpi) and stained with anti-UIS4 and anti-MSP1 antibodies. Quantification of EEF numbers and area revealed no significant differences between *Pb*Cls KO and WT-GFP parasites (Fig 5F–5I). Analysis of anti-MSP1-stained EEFs revealed reduced nuclear segregation and impaired differentiation into merozoites (Fig 5G, 5J and 5K). Quantification of liver-stage forms further demonstrated a significant accumulation of cytomere forms and reduction in mature merozoites in *Pb*Cls KO parasites compared to WT-GFP (Fig 5K). To further investigate this, we quantified detached cells formation at 65 and 80 hpi and consistently observed detached cells in WT-GFP parasites, but not in the *Pb*Cls KO parasites (Fig 5L). To functionally assess infectivity, detached cells from WT-GFP cultures or equivalent volumes of *Pb*Cls KO culture supernatants were injected into Swiss albino mice (n = 5 per group). All mice receiving WT-GFP-derived detached cells developed blood-stage infections, whereas none of the mice injected with *Pb*Cls KO supernatants became patent (Table 2). We then assessed mitochondrial biogenesis in *Pb*Cls KO EEFs. Mitochondrial branching was impaired in *Pb*Cls KO parasites compared to the extensive branching seen in WT-GFP parasites. Quantification of mitochondrial branching in EEFs confirmed a significant reduction in *Pb*Cls KO parasites. While a small subset of *Pb*Cls KO EEFs at 40 hpi exhibited mitochondrial morphology comparable to WT-GFP, none of the *Pb*Cls KO EEFs analyzed at 65 hpi displayed normal mitochondrial branching (Fig 6A and 6B). Finally, parasitophorous vacuole membrane (PVM) integrity at the late liver stage was analyzed using anti-UIS4 staining. At 65 hpi, WT-GFP parasites showed PVM breakdown consistent with maturation and egress, whereas *Pb*Cls KO EEFs retained intact PVMs (Fig 6C and 6D), further supporting a defect in maturation and egress. These results collectively demonstrate that *Pb*Cls is dispensable for sporozoite invasion and early liver-stage development but is critical for mitochondrial biogenesis, hepatic merozoite maturation and successful transition to the blood stage.

**Table 2:** Infectivity of detached cells in Swiss mice.

Experiment	Parasites	Number of merosomes injected	Mice positive/mice injected	Pre-patent period (days)
1	WT GFP	10	3/3	4.0
	*Pb*Cls KO	Supernatant	0/5	N/A
2	WT GFP	10	3/3	4.0
	*Pb*Cls KO	Supernatant	0/5	N/A
3	*Pb*Cls KO	Supernatant	0/5	N/A

## Discussion

Maintenance of cellular function across life cycle stages of *Plasmodium* parasites requires a balance between import of membrane phospholipids and their de novo synthesis [1,42]. CL is a specialized phospholipid of the IMM in eukaryotes with demonstrated roles in mitochondrial biogenesis and function. Our localization of *Pf*Cls, the final enzyme of the CL synthesis pathway, to the mitochondrion of parasite asexual blood stages is indicative of its role in mitochondrial CL formation de novo. In addition to its mitochondrial presence in gametocytes, *Pf*Cls signals towards the cellular periphery and the detection of CL in the PPM in late gametocyte stages suggest its extra-mitochondrial function. CL presence outside mitochondria is not unknown. In addition to its presence in yeast and plant peroxisomes and in the nuclear membrane of *Tetrahymena* [9,10], relocation of mitochondrial CL to the plasma membrane and membranes of other organelles is reported during apoptosis in mouse liver cells [43]. The presence of *Pf*Cls at the cytosolic periphery in gametocytes suggests its direct role in formation of CL incorporated into the PPM. *Pf*Cls is highly expressed in stage V gametocytes compared to early gametocytes and asexual blood stages [44]. The lipid composition of mature gametocytes is reported to differ widely from asexual blood stages and early gametocytes with increase in cholesterol and sphingomyelin, which are regulators of membrane fluidity, in late gametocytes [3,7]. Although CL abundance has not been compared in these studies, our results suggest a role of CL in the mature gametocyte plasma membrane as well as mitochondria.

Increased CL levels in *E. coli* expressing *Pf*Cls and the decrease in CL abundance upon genetic ablation of *Cls* in *P. berghei* confirms its role in CL biosynthesis. The accompanying decrease in PE in *Pb*Cls KO parasites suggests an additional function of *Plasmodium* Cls in PE formation. Since PE is also an important part of mitochondrial membranes along with CL, the decrease in mitochondrial membrane potential in KO lines might correlate to the combined effect of the two phospholipids on mitochondrial function. PE formation by bacterial Cls is a feature reported for the PLD-type bifunctional Cls from *Xanthomonas campestris* and *P. syringae* [18,41]. While formation of CL is the primary function of these bacterial enzymes, they also synthesize PE from CDP-DAG and EA (Fig 1B). The latter function of the *P. syringae* enzyme was confirmed in vivo but was not observed with the purified enzyme, indicating that additional cellular component(s) were required for such PE biosynthesis [18]. We also failed to detect PE formation from CDP-DAG and EA with both purified *Ps*Cls and *Pf*Cls, suggesting a role for additional factors. In eukaryotes, PE is mainly synthesized in the endoplasmic reticulum (ER) from DAG and EA via the CDP-EA (Kennedy) pathway, and by decarboxylation of PS catalyzed by phosphatidyl serine decarboxylase (Psd) enzymes in the mitochondria [45]. The Kennedy pathway enzymes are proposed to be essential for *P. berghei* blood stages [46,2], and an ER-localized type-1 Psd is characterized in *P. falciparum* [47]. The possible additional involvement of *Plasmodium* Cls in PE formation using CDP-DAG requires further exploration with lipid radio-labelling experiments in vivo. The significant reduction observed across both PE and PS molecular species in the *Pb*Cls KO can result from a two-way effect. Not only can reduced PS levels affect PE formation catalysed by Psd, lowering of PE might in turn impact PS formation via the DAG-independent PS synthase E2 (PSSE2), an enzyme which catalyzes base exchange of serine with PE to form PS in mammals and plants and is reported in *Plasmodium* [48,49]. Although PE and PC synthesis are linked by the Kennedy pathway [46,48], reduction in PC levels in the *Pb*Cls KO were not statistically significant.

Recombinant *Pf*Cls catalyzed formation of PG from PE and glycerol, which is also an enzymatic property of *E. coli* ClsB and *Ps*Cls [18,22] (S9 Fig). Incubation with CDP-DAG as lipid substrate formed PA, showing that *Pf*Cls has PLD activity and can hydrolyze CDP-DAG. Bacterial PLD-type enzymes which lack a CDP-DAG catalytic motif can utilize CDP-DAG as substrate as reported for *Ps*Cls and *E. coli* phosphatidyl serine synthase [50]. This is a property shared by *Pf*Cls, which also binds well to CDP-DAG vesicles in vitro. The inability of recombinant *Pf*Cls to make detectable CL from 2PG or PG + PE might reflect the requirement of *Pf*Cls to be part of a protein complex in vivo, as seen in our protein crosslinking experiment, for manifestation of this activity. *T. brucei* bacterial-like Cls is also reported to be part of an ~ 700 kDa mitochondrial protein complex [17].

De novo CL synthesis in yeast and metazoans is followed by CL remodelling by two-step deacylation-reacylation by phospholipases and acyltransferases/transacylases, or by acyl remodelling through a single transacylation mediated by transacylases [51]. Tafazzin, the transacylase that remodels CL is not identified in alveolates including *Plasmodium* spp. [20]. Phospholipases iPLA2β or iPLA2γ have a role in CL remodelling in *Drosophila* and mammalian cells [52,53]. A recent study has identified a *P. falciparum* patatin like phospholipase 2 (*Pf*PNPLA2) which localizes to the mitochondrion as a possible CL remodeller [29]. Disruption of this protein is associated with moderate change in composition of CL to shorter chained and more saturated species, with reduction in asexual blood stage growth and effect on gametocyte maturation. The asexual growth of *Pb*Cls KO parasites also slows down compared to WT parasites, although Cls does not seem to be essential for parasite survival at this stage. Genome-wide transposon mutagenesis has identified Cls as ‘essential’ for blood stages in *P. falciparum* and *P. knowlesi* [54,55], both of which can infect mature erythrocytes lacking mitochondria. *P. berghei*, on the other hand, has a strong preference for immature RBCs (reticulocytes) which retain remnant organelles, including mitochondria, and may provide a limited pool of host-derived phospholipids such as CL or its precursors [56]. Intra-erythrocytic parasites are known to exhibit metabolic plasticity, enabling uptake and remodelling of phospholipids from the host [56,57]. It is possible that the CL requirement in *Pb*Cls KO can be partly met by scavenging host reticulocyte CL resulting in a more modest phenotypic effect. CL scavenging from the host cell has been reported in the related apicomplexan *Toxoplasma gondii* where virulent Type I parasites synthesize CL de novo and also use host fibroblast CL transported across the selectively permeable parasitophorous vacuole membrane [58]. Furthermore, unidentified salvage or remodelling pathways contributing to CL homeostasis remains a possibility. No other bacterial- or eukaryotic-type Cls homolog is identifiable in *Plasmodium* genomes, supporting the likelihood of indirect compensation rather than redundancy. Consistent with known effects of CL depletion on mitochondrial ETC function and interaction of respiratory chain complex proteins with CL reported in *T. gondii* [59], *Pb*Cls-depleted blood stage parasites exhibit partial mitochondrial membrane depolarization and have a lower basal oxygen consumption rate. CL deficiency might also adversely affect *Plasmodium* mitochondrial carrier proteins with CL-dependent transport activities [60]. The impaired parasite proliferation in *Pb*Cls KO asexual blood stages is further reflected in significantly reduced gametocytemia at day 3, with gametocyte levels recovering by day 4 post-infection to approach numbers in WT parasites.

The dispensability of *Pb*Cls for parasite development in the mosquito vector suggests that de novo CL synthesis requiring *Pb*Cls is not essential in the parasite ookinete, oocyst and sporozoite stages, and may be compensated for by other phospholipids such as PA and PE, or by altered lipid metabolism and adaptation in these stages. Parasites in the mosquito are known to exploit host-derived lipids and exhibit stage-specific remodelling of membrane composition, which could compensate for CL in *Pb*Cls KO parasites [61]. De novo CL synthesis, however, seems to be important for late liver stage development as *Pb*Cls KO parasites have defects in mitochondrial biogenesis and maturation of hepatic merozoites, and exhibit delayed transition from the liver to the blood stage. Parasite replication in hepatocytes enlarges the parasite vacuolar membrane and generates thousands of merozoites thus generating a high demand for lipids. This is primarily met by fatty acids synthesized by the FASII pathway of the apicoplast which are central to lipoic acid biosynthesis and as precursors for membrane lipids in EEFs [62,63]. Growing *P. berghei* EEFs also use phospholipids and cholesterol derived from host hepatocytes which have elevated levels of sphingolipids, cholesterol, neutral lipids and phosphatidylcholine (PC) [64,65]. In maturing liver stages, the mitochondrion is very large with complex branching and has tubular cristae [24,66,67]. There is also an increased expression of genes of the mETC and the TCA cycle indicating high mitochondrial metabolic activity [63,68–71]. CL deficiency in *Pb*Cls KO parasites inhibits mitochondrial branching in late EEFs as observed in this study. Whether it also perturbs formation of mitochondrial cristae, assembly of respiration supercomplexes and protein import remains to be explored.

Our results identify a bacterial-type Cls from human and rodent malaria parasites as important for asexual blood-stage replication and liver-stage maturation. Its role in maintenance of optimal mitochondrial function is highlighted by the effect of genetic ablation of *Pb*Cls on mitochondrial membrane potential and basal respiration in blood stages and in mitochondrial branching in EEFs. Novel insights are provided into its dual mitochondrial and cytosolic localization in late gametocytes and biochemical analysis of its PLD-type Cls activity. Taken together, these results provide the first characterization of an enzyme for biogenesis of an important mitochondrial phospholipid in the malaria parasites.

## Materials and methods

### Ethics statement

All animal procedures were performed in accordance with the guidelines approved by the Institutional Animal Ethics Committee of Council of Scientific and Industrial Research-Central Drug Research Institute, India (Approval no: IAEC/2018/03 and IAEC/2025/124).

### Sequence analysis and phylogeny

Sequences of *Pf*Cls and its homologs were obtained from OrthoMCL in PlasmoDB [72] and NCBI database (www.ncbi.nlm.nih.gov). Multiple sequence alignments were performed by Clustal Omega [73]. Transmembrane domain prediction was made using TMHMM [74] and mitochondrial targeting score of *Pf*Cls was obtained by MitoProt II [75]. Active site motifs and PLD domain were identified via ScanProsite [76].

A maximum-likelihood unrooted phylogenetic tree was constructed for a total of 19 protein sequences (S2 Table) using LG + R + F model by ATGC: SMS (Smart Model Selection) in PhyML 3. 0 [77], with 1000 bootstrap replicates.

### Parasites, mosquitoes, and mice

*P. falciparum* 3D7 (source: MR4, BEI Resources, USA) were cultured in human RBCs (approval CDRI/IEC/2017/A4 by Institutional Ethics Committee- Human Research) in RPMI-1640 medium (HEPES-modified) (Sigma Aldrich, USA) supplemented with 0.2% sodium bicarbonate, 1% glucose, 100 μM hypoxanthine, gentamycin (25 μg/ml) and 0.5% Albumax II (Invitrogen, USA). *P. falciparum* 3D7 gametocytes (stage I to stage V) were obtained by initial culture at 1% parasitemia and 4% hematocrit for 12–14 days, with supplementation of RPMI culture medium each day without adding fresh RBCs [78].

Parasites were synchronized by treatment with 5% w/v D-sorbitol (Sigma-Aldrich, USA). Parasite lysate was prepared by releasing parasites from RBC by 0.5% saponin lysis, followed by washing with cold phosphate buffered saline (PBS) containing protease inhibitor cocktail (Sigma-Aldrich, USA). The parasite pellet was treated with RIPA buffer (50 mM Tris-HCl pH 7.5, 300 mM NaCl, 0.1% Triton X-100, 0.1% SDS, 0.5% sodium deoxycholate) and sonicated for 5 min at 25% amplitude.

*P. berghei* ANKA (MRA-311) and *P. berghei* ANKA GFP (MRA-867, 507 m6cl1; WT-GFP) strains were obtained from BEI Resources (USA). *Anopheles stephensi* mosquitoes were reared under controlled conditions at 28°C and 80% relative humidity. The parasite life cycle was maintained using 6–8 week-old Swiss albino mice. For in vivo sporozoite infectivity assays, 4–6 week-old C57BL/6 mice were used.

### Recombinant protein expression and purification

The 1.6 kb gene segment encoding *Pf*Cls (1–552 amino acids) was PCR-amplified using specific primers (S3 Table) with *P. falciparum* 3D7 genomic DNA as template and cloned into EcoRI and HindIII sites in the pET23a(+) expression vector carrying a 6X-His tag. The clone was confirmed by DNA sequencing. To purify *Pf*Cls, *E. coli* BL21-Codon plus (DE3) were transformed with pET23a(+)-*Pf*Cls and grown in LB media supplemented with ampicillin (100 μg/ml) and chloramphenicol (25 μg/ml) at 37˚C until OD_600_ of 0.6 was reached. Culture was then induced with 0.5 mM IPTG at 20˚C for 16 h. The cells were harvested and the induced culture pellet was re-suspended in lysis buffer with an ionic detergent (50 mM Tris-HCl, pH 8.0, 250 mM NaCl, 10% glycerol, 1mM PMSF, 0.3% N-lauryl sarcosine) followed by 30 min incubation at 4 ˚C. Cells were lysed by sonication and centrifuged at 10,733 × g for 45 min at 4˚C. The clarified cell lysate was collected and loaded on pre- equilibrated Ni-nitrilotriacetic acid (Ni-NTA) Superflow (Qiagen, Germany) affinity column followed by sequential washing with buffer (50 mM Tris-HCl, pH 8.0, 250 mM NaCl, 10% glycerol) containing 10 mM and 20 mM imidazole and elution with buffer containing 300 mM imidazole. The eluted protein was checked by SDS-PAGE and western blotting with anti-6XHis Ab (Santa Cruz Biotechnology, USA). After concentration and buffer exchange using Centricon filters (Merck, USA) the protein was quantified by Bradford assay (Sigma-Aldrich, USA). *P. syringae* Cls [18] was purified from the pET28b-PSPTO_0095 construct by Ni-NTA purification as described for *Pf*Cls above.

Affinity-purified *Pf*Cls was injected in Superdex 75 (S75) 10/300 GL column (GE Healthcare, USA) on an AKTA (UNICHROMAT 1500; GE Healthcare, USA) for size exclusion chromatography. The column was pre-equilibrated with buffer (50 mM Tris-HCl pH 8.0, 200 mM NaCl, 5% glycerol). Different fractions were collected, concentrated and separated by SDS-PAGE. For sizing, standard protein markers conalbumin (75 kDa), ovalbumin (43 kDa), carbonic anhydrase (29 kDa) and ribonuclease A (13.7 kDa) (GE Healthcare, USA) were separately fractionated on the same column.

Solution peptide mass spectrometry of purified *Pf*Cls was carried out at Valerian Chem Private Limited, India. Briefly, the sample was first reduced with 5 mM TCEP and then alkylated with 50 mM iodoacetamide. After digestion with Trypsin for 16 h at 37°C, the mixture was purified using a C18 silica cartridge and concentrated. The dried pellet was re-suspended in buffer A (2% acetonitrile, 0.1% formic acid). Mass spectrometric analysis of the peptide mixture was performed on an Easy-nlc-1000 system coupled with an Orbitrap Exploris 480 mass spectrometer (Thermo Fisher Scientific, USA). The mass/charge values were analysed through Thermo Proteome Discoverer (v2.5) against the Uniprot Reference database. For Sequest and Amanda search, the precursor and fragment mass tolerance were set at 10 ppm and 0.02 Da, respectively.

For generation of antibodies against *Pf*Cls, the C-terminal segment (330–590 amino acids) of *Pf*Cls (cloned in pET21d(+)-*Pf*Cls-CTD, gift from Dr. Niti Kumar) was expressed in *E. coli* BL21-Codon plus (DE3). Since the protein appeared in the insoluble fraction, inclusion bodies of *Pf*Cls-CTD were purified by washing with buffer (50 mM Tris-HCl, pH 8.0, 250 mM NaCl) containing 0.05% TritonX-100. The protein was electrophoresed on 12% SDS-PAGE and stained with Coomassie G-250. The 30.8 kDa *Pf*Cls-CTD protein band was cut from the gel followed by destaining and electroelution in 10 mM EDTA in a 14 kDa-cutoff dialysis membrane.

### Circular dichroism spectroscopy

CD spectrum of *Pf*Cls (5 µM protein in 20 mM Tris, pH 8.0,150 mM NaCl,10% glycerol) was recorded at 25°C in a CD spectropolarimeter (model JASCO-1500). Spectral scans with a total of three accumulations were recorded over a range of 190–250 nm. Scanning speed was set 50 nm/min with a 1 cm path length.

### Raising of polyclonal antisera

Purified *Pf*Cls-CTD was dialyzed in 1xPBS and to immunize rabbit (New Zealand White) (Institutional Animal Ethics Committee approval no. IAEC/2007/126/(292–5/18) Ren11). Prior to immunization, serum from the rabbit was checked against parasite lysate to confirm absence of cross-reacting antibodies. An emulsion of equal volume of protein (300 µg) and Freund’s complete adjuvant was injected subcutaneously as primary immunization. The first booster dose of *Pf*Cls (100 µg) in Freund’s incomplete adjuvant was given 28 days post-immunization with the second booster dose after 10 days of the first booster. The rabbit was bled to obtain anti-*Pf*Cls polyclonal antisera after 10 days of the second booster. The antiserum (1:1000) was used to detect *Pf*Cls in *P. falciparum* lysate in western blots with HRP-conjugated goat anti-rabbit IgG (1:5000, Sigma Aldrich, USA) as secondary Ab. The blots were developed using the ECL kit (Merck Millipore, USA).

### Differential membrane fractionation

Membrane fractionation was carried out as described by Tanveer et al. (2013) [79]. *P. falciparum* trophozoites were incubated in lysis buffer (50 mM Tris-Cl pH 7.4, 2 mM EDTA, 1X protease inhibitor cocktail) for 30 min on a tube rotator and centrifuged at 26,500 × g for 40 min to separate soluble proteins from the insoluble fraction. The Tris-insoluble fraction was resuspended in carbonate buffer (0.1 M sodium carbonate pH 11, 1 mM EDTA), incubated for 30 min followed by centrifugation at 26,500 × g for 40 min to extract extrinsic membrane proteins in the supernatant. To extract intrinsic membrane proteins from the carbonate insoluble pellet, the pellet was treated with 2% Triton X-100 in PBS for 30 min. The soluble fraction obtained after centrifugation at 26,500 × g for 40 min represents integral membrane proteins while the pellet fraction has insoluble protein. The pellet was suspended in 1X PBS in the same volume as the supernatant. Equal volume of sample from all fractions were loaded on SDS-PAGE. For western blotting, the membrane was cut and probed separately with anti-*Pf*Cls antisera (1:1000) and anti β-actin antibody (1:50000, Sigma Aldrich, USA).

### Chemical cross-linking

Parasite proteins were cross-linked in vivo with DSP (dithiobis(succinimidyl propionate); Merck, Germany) as described in [80]. Briefly, infected RBCs were resuspended in PBS containing 2 mM DSP at room temperature. After incubation for 30 min, the reaction was stopped by addition of quenching solution (final concentration: 25 mM Tris-Cl pH 7.5, 100 mM NaCl) and incubated for 15 min. Parasites were released by saponin lysis, resuspended in NRSB buffer [0.05 M Tris-Cl pH 6.8, 10% glycerol, 2 mM EDTA, 2% SDS, bromophenol blue] with different concentrations of DTT, followed by incubation at 80 °C for 5 min. The samples were electrophoresed in a 6–10% gradient SDS-PAG followed by western blotting with anti-*Pf*Cls Abs (1:1000).

### Thermolysin protection assay

Thermolysin protection assay was performed as described previously [81,82]. *P. falciparum* 3D7 parasites (at trophozoite stage) were resuspended in assay buffer (50 mM HEPES-NaOH, pH 7.4, 0.5 mM CaCl_2_, 300 mM sorbitol) containing no detergent (control), 0.05% digitonin or 0.05% digitonin +10 mM EDTA. After incubation at room temperature for 10 min, thermolysin (Sigma Aldrich, USA) was added to a final concentration of 25 µg per 1 mg of parasite protein. Reactions were incubated at room temperature for 35 min and 15 min on ice. The reactions were stopped by addition of 10 mM EDTA. Western blotting was done using anti-*Pf*Cls sera (1:1000), mouse anti-*Pf*HU sera (1:250) [83] and anti-α-tubulin Ab (1:500) (Sigma-Aldrich, USA).

### Immunofluorescence assay and confocal microscopy

*P. falciparum* infected RBCs at the trophozoite, schizont or gametocyte stages were processed for immunofluorescence assay. For mitochondrial staining, cells were incubated in 50 nM MitoTracker Red CMXRos (Invitrogen, USA,) for 30 min at 37°C. Parasite cells were fixed in 1XPBS containing 0.0075% glutaraldehyde (prepared in 4% paraformaldehyde) for 30 min, followed by permeabilization with 0.2% Triton X-100 for 20 min at room temperature. Permeabilized cells were washed with 1XPBS and blocked in 3% BSA (in PBS). Cells were then incubated overnight at 4°C with primary antisera *Pf*Cls (1:50), mouse anti-*Pf*HU (1:100) [83] and anti-tubulin Ab (1:250; Sigma-Aldrich, USA). Alexa Fluor 568-tagged anti-rabbit Ab and Alexa Fluor 488-tagged anti-mouse Ab (1:1000; Invitrogen, USA) were used as secondary antibodies. Alexa Fluor 514-tagged anti-rabbit (1:1000; Invitrogen, USA) was used as the secondary antibody for *Pf*Cls in MitoTracker labelled cells. DAPI (0.2 µg/ml) was used for nuclear staining. Cells were seeded on poly L-lysine coated coverslips, allowed to adhere for 2 h, washed three times with 1XPBS, and coverslips were mounted in mounting media (Oncogene Research Products, USA). Samples were scanned under a 63X or 100X/1.4 oil immersion objective in Leica-SP8 confocal microscope. Co-localization was analyzed by the Leica Las X software.

For IFAs with *P. berghei*, parasites at the blood and liver developmental stages were harvested and subjected to IFA as previously described [84]. Briefly, parasites were fixed in 4% paraformaldehyde for 20 min at room temperature. Blood stage smears were permeabilized with 0.1% Triton X-100 (Sigma-Aldrich, USA) for 10 min at room temperature, while liver stage parasites were permeabilized using chilled methanol. Following permeabilization, samples were blocked with 1% BSA for 1 hour at room temperature. Samples were then incubated with anti-*Pf*Cls serum (1:100 dilution) for 1 h at room temperature. After washing, samples were incubated with Alexa Fluor 594-conjugated anti-rabbit IgG secondary antibody (1:1000 dilution; Invitrogen, USA). Parasite nuclei were stained with Hoechst 33342.

For IFA in *E. coli*, bacteria transformed with pET23a(+)-*Pf*Cls or the vector alone were fixed with 2.8% formaldehyde and 0.04% glutaraldehyde in1XPBS for 15 min at room temperature and then permeabilized with 0.1% Triton-X-100 for 45 min at room temperature. The cells were washed three times with 1XPBS and incubated in 100 µg/ml lysozyme and 5 mM EDTA for 45 min at room temperature [85]. Cells were incubated with anti-6XHis Ab (1:100, mouse monoclonal; Santa Cruz Biotechnology, USA) overnight at 4°C, followed by incubation with secondary antibody Alexa Fluor 488-tagged anti-mouse Ab (1:1000; Invitrogen, USA) for 2 h at room temperature. DAPI (0.2 µg/ml) was used for nucleoid staining. Cells were seeded on poly L-lysine coated coverslips and scanned on Leica-SP8 confocal microscope under 100X/1.4 oil immersion objective. Identical laser intensity, gain and line accumulation settings were set for comparison of signals in vector alone and *Pf*Cls-expressing cells.

### Protein lipid overlay assay

Protein lipid overlay assay (dot blot) was performed as described by Lu et al. (2020), and Aktas & Narberhaus (2009) [86,87]. Phospholipids PE (18:1(ρ9-Cis) PE(DOPE)1,2-dioleoyl)-sn-glycero-3-phosphoethanolamine, PG (18:1(ρ9-Cis) PG 1,2-dioleoyl-sn-glycero-3-phospho-(1’-rac-glycerol) (sodium salt), CDP-DAG (18:1 CDP DG 1,2-dioleoyl-sn-glycero-3-(-cytidine diphosphate) (ammonium salt) and CL (18:1,Cardiolipin 1’,3’-bis[1,2-dioleoyl-sn-glycero-3-phospho]-glycerol (sodium salt) (Avanti Polar Lipids, USA) were tested for binding *Pf*Cls. 10 µl of different lipid concentrations in buffer (50 mM Tris-Cl pH 8.0, 100 mM NaCl) were spotted onto nitrocellulose membrane (Cytiva, USA), allowed to dry at room temperature for 1 h and blocked with 5% skimmed milk for 3 h. The membrane was washed three times with 1xPBS followed by incubation with 5 µM *Pf*Cls overnight at 4°C with gentle rocking. Membrane was washed three times over 30 min in PBST (PBS containing 0.5% Tween 20) followed by detection of bound *Pf*Cls using anti-6XHis antibody (1:5000) and HRP-conjugated anti-mouse secondary antibody (1:5000; Sigma-Aldrich, USA). Membrane was washed three times with PBST over 30 min and the signal detected by chemiluminescence (ECL kit, Merck Millipore, USA). Signals were quantified using Image Quant TL (Cytiva, USA).

### Liposome co-sedimentation assay

Liposomes were prepared as described by Danne et al. [88]. Briefly, CL, PG, PE and CDP-DAG were dissolved in chloroform and dried under nitrogen gas flow in glass tubes. 1 ml of rehydration buffer (50 mM Tris, pH 8.0, 100 mM NaCl) was added to prepare lipids at a final concentration of 1.5 mM and incubated for 1 h at room temperature. The suspension was bath-sonicated for 5 min. The sonicated lipid suspension was extruded 10 times through an Avanti mini-extruder with 400 nm polycarbonate filter (Avanti Polar Lipids, USA). The particle size of the extruded lipid suspensions was measured by dynamic light scattering using a Zetasizer Nano ZS (Malvern Panalytical, UK). Purified recombinant *Pf*Cls was buffer exchanged in rehydration buffer. The interaction between the *Pf*Cls and liposomes was measured by incubating 10 µM *Pf*Cls with 0.5 mM liposomes of each phospholipid in rehydration buffer at room temperature. BSA (2%) was added to all reaction mixtures to prevent nonspecific interaction of *Pf*Cls with liposomes. Negative control tube had *Pf*Cls without a phospholipid. Liposome-bound and free proteins were separated by ultracentrifugation at 82,368 × g (Beckmann Optima XPN-100 ultracentrifuge, fixed-angle rotor 70.1Ti) for 1.5 h at 4°C. The supernatant and pellet fractions were separated; to prevent disruption of the pellet, 10 μl volume was left in the pellet fraction. Equal volumes (10 μl) of the fractions were analyzed for the presence of *Pf*Cls by western blotting using anti-6XHis antibody.

### CL detection by NAO staining

*E. coli* cells transformed with pET23a(+) or pET23a(+)-*Pf*Cls were induced with 0.5 mM IPTG for 12 h at 20°C and incubated with 250 nM of 10-N-nonyl acridine orange (NAO) (Invitrogen, USA) for 1 h at 37°C with gentle shaking [36,41]. Cells were layered on poly L-Lysine coated coverslips, allowed to adhere for 1 h at room temperature, washed three times with 1XPBS, and coverslips were mounted in mounting media. Samples were scanned on Leica-SP8 confocal microscope under 100X/1.40 oil objective. NAO fluorescence was detected at 488 nm/528–563 nm for green fluorescence and 488/600–650 nm for red fluorescence. Identical laser intensity and gain settings were used to compare signals in vector alone and *Pf*Cls sets. Line accumulation was set at 1 for green fluorescence and 16 for red fluorescence. Red fluorescence mean intensity at the cell poles was quantified by LAS X Office software.

For CL detection in parasites, iRBCs were incubated with 500 nM of NAO and 50 nM MitoTracker Red CMXRos at 37°C for 1 h with gentle shaking. Cells were seeded on poly L-Lysine coated coverslips, washed three times with 1XPBS and mounted in mounting media. Fluorescence detection was carried out with Leica-SP8 confocal microscope under 63X/1.4 oil immersion objective. NAO fluorescence was detected at 488 nm/528–563 nm.

### *Pf*Cls in vitro activity assay

Micelle preparation for generating substrates to assay recombinant *Pf*Cls activity was carried out as described by Aktas and Narberhaus [87]. Briefly, 10 mg/ml stock solutions of PG, PE, CDP-DAG and CL in chloroform: methanol (1:1) were evaporated with nitrogen gas under vacuum. Dried phospholipids were resuspended in activity buffer (50 mM NaH_2_PO_4_, 300 mM NaCl, DDM [4CMC], pH-8.0) and Triton X-100 in the Triton: phospholipid molar ratio of 2:1 to solubilize the phospholipids.

For assaying activity with different phospholipid micelle substrates, 5 µM purified recombinant *Pf*Cls was incubated with 0.4 mM CDP-DAG, 0.4 mM CDP-DAG with 0.4 mM EA, 0.5 mM PE with 10% glycerol, 0.5 mM PG, or 0.5 mM each of PG and PE for 16 h at 30°C. The total reaction volume was 100 µl. 1 µM of purified *P. syringae* Cls was used as positive control in the reactions. Total lipids were extracted and analyzed via TLC as described by Vasilopoulos et al. [18]. Lipids were extracted using the Bligh and Dyer method [89]. 375 µl of chloroform: methanol (1:2) was added to the 100 µl reaction volume and vortexed for 30 sec followed by addition of 125 µl of chloroform and mixing by vortexing.125 µl of deionized water was added to separate the organic and aqueous phases. Samples were centrifuged at 100 × g for 10 min. The organic phase was recovered in a fresh tube followed by washing with 125 µl of deionized water. The organic phase was recovered after centrifugation and dried under N_2_ gas flow. Dried lipids were resuspended in 20 µl chloroform: methanol (1:1) before spotting on a TLC plate. 5 µl of isolated lipids from activity reactions was spotted on TLC plate (HPTLC silica gel, Merck, USA). Chloroform: methanol: glacial acetic acid: water (3.2:0.2:0.5:0.1 [v/v]) was used as the mobile phase for lipid separation. The TLC chamber was pre-saturation with the mobile phase. After chromatography, the TLC plate was dried at 100°C followed by development with 10% phosphomolybdic acid (Himedia, India) or molybdenum blue spray reagent (Sigma-Aldrich, USA) [18,90].

### Lipid extraction from *E. coli* and two-dimensional TLC (2D-TLC)

*E. coli* cells transformed with pET23a(+) or pET23a(+)-*Pf*Cls were induced with 0.5 mM IPTG and grown to stationary phase (OD_600nm_ of ∼2.0) in 100ml LB media. Lipid extraction was performed by acidic Bligh and Dyer method [89]. Cells were pelleted and resuspended in 2 ml of 0.1 N HCl followed by addition of 5 ml methanol and 2.5 ml chloroform to form a single-phase solution comprise of chloroform/methanol/0.1 N HCl [1:2:0.8 (vol/vol)]. After incubation at room temperature with gentle mixing, 2.5 ml each of 0.1 N HCl and chloroform were added to convert the single phase to a two-phase solution made up of chloroform/methanol/0.1N HCl [2:2:1.8 (vol/vol)]. The solution was centrifuged at 3000 × g for 25 min at room temperature. The lower organic phase was collected in separate tubes and dried under nitrogen stream. The dried lipids were resuspended in 100 μl of chloroform/ methanol (2:1) and sonicated for 1 min in bath sonicator. 5 µl of each sample was spotted on TLC plate and solvent mixtures of chloroform:methanol:water (65:25:4) and chloroform:methanol:acetic acid:water (90:15:10:3,5) were used as mobile phase for the first and second dimension, respectively. TLC plates were dried at 100°C and developed with 10% phosphomolybdic acid [23,31]. PG, PE and CL were used as phospholipid standards. Phospholipids were quantified by densitometry via Image Quant TL (Cytiva, USA).

### Generation of *Pb*Cls knockout and complement parasite lines

Targeted disruption and complementation of *Pb*Cls in *P. berghei* ANKA were performed as previously described [91]. For gene disruption, two homology regions were PCR-amplified from *P. berghei* genomic DNA: a 570 bp upstream fragment (F1) using primers 2101/2102 and a 569 bp downstream fragment (F2) using primers 2103/2104. These fragments were sequentially cloned into the pBC-GFP-hDHFR:yFCU vector. The resulting knockout construct was linearized with XhoI and AscI, and was transfected into purified schizonts using basic parasite nucleofector kit (Lonza) as described previously [92]. Transfected parasites were selected with pyrimethamine, and genomic DNA from drug-resistant populations was screened for correct integration by diagnostic PCR using primer pairs 2183/1225 (5′ integration) and 1215/2184 (3′ integration). Clonal lines were obtained by limiting dilution and screened for the absence of the endogenous *Pb*Cls locus using primers 2206/2207. For genetic complementation, a 1,809 bp genomic fragment encompassing the full-length *Pb*Cls coding region and flanking sequences was amplified using primers 2101/2104 and reintroduced into the knockout line. Transfectants were selected with 5-fluorocytosine (Sigma-Aldrich, USA) to select against the yFCU marker. Restoration of the wild-type *Pb*Cls locus was confirmed by PCR using primers 2206/2207. All primer sequences are provided in S3 Table.

### Blood growth assay

To investigate the role of *Pb*Cls during the asexual blood stages of *P. berghei*, female Swiss albino mice (5 mice per group) were intravenously injected with parasitized red blood cells (pRBCs) at 0.5% parasitemia. Parasitemia was monitored daily by preparing Giemsa-stained blood smears.

### Mitochondrial membrane potential assay

To assess mitochondrial membrane potential, equal volumes of parasitized blood were collected from each group. Parasites were incubated with 50 μM carbonyl cyanide m-chlorophenyl hydrazone (CCCP), dissolved in DMSO and diluted in 1X PBS, at 37°C for 1 h as a control for mitochondrial depolarization. Following incubation, samples were washed twice with 1X PBS. WT-GFP, *Pb*Cls KO, and CCCP-treated parasites were then stained with MitoTracker Deep Red FM at 37°C for 30 min. After staining, cells were washed once with 1X PBS and counterstained with Hoechst 33342 to visualize nuclei. Fluorescence intensity was quantified using Fiji (ImageJ). Mean fluorescence values were calculated per parasite and normalized across biological replicates.

### Assessment of *Pb*Cls KO in mosquito stage development and sporozoite infectivity

To assess the role of *Pb*Cls during mosquito-stage development, *A. stephensi* mosquitoes were allowed to feed on infected Swiss mice, and parasite development at various stages was analyzed as described previously [93]. At 24 h post-blood feeding, mosquito midguts were dissected, and ookinetes were enumerated by light microscopy. On day 14 post-feeding, midguts were dissected, and the number of oocysts per midgut was counted using a fluorescence microscope. Between days 18 and 23 post-feeding, salivary glands were dissected, homogenized in RPMI-1640 medium, and sporozoites were quantified using a hemocytometer.

To assess the in vivo infectivity of sporozoites, C57BL/6 mice were intravenously injected with 5,000 salivary gland sporozoites. Parasite development was monitored by examining Giemsa-stained thin blood smears. The pre-patent period was determined as the time from injection to the first detection of blood-stage parasites.

### In vitro EEF development and IFA

For in vitro EEF development, HepG2 cells were cultured and infected with salivary gland sporozoites. Infected cultures were harvested at various time points, fixed with 4% paraformaldehyde, washed twice with 1 × PBS, permeabilized with methanol, and blocked in 1% BSA (Sigma-Aldrich, USA) for 30 min. Primary antibodies anti-UIS4 (diluted 1:1,000) [94] and anti-MSP1 (diluted 1:5,000) [95] were diluted in blocking solution and incubated for 1 h at room temperature. To reveal UIS4 and MSP1 signals, slides were incubated with Alexa-Fluor 594 conjugated anti-rabbit IgG (diluted 1:000; Invitrogen, USA) and Alexa-Fluor 594 conjugated anti-mouse IgG (diluted 1:1000; Invitrogen, USA), respectively. Nuclei were stained with Hoechst 33342. Nuclei were stained with Hoechst 33342, and slides were mounted using prolong diamond antifade reagent (Invitrogen, USA). Images were captured using a 100X oil immersion objective on an Olympus BX61WI confocal microscope. To assess detached cells formation, 1 × 10⁵ HepG2 cells were seeded in a 24-well plate and infected with 30,000 sporozoites. Culture supernatants were collected at 64 and 80 hpi, and detached cells were quantified using a hemocytometer. To evaluate the infectivity of detached cells, Swiss albino mice were intravenously injected with harvested detached cells or culture supernatant. The onset of blood-stage infection was monitored using Giemsa-stained blood smears. To assess mitochondrial morphology in late liver-stage EEFs, samples were harvested and processed for IFA using an anti-IscU Ab (1:100 dilution) [96]. Signals were revealed using Alexa Fluor 594-conjugated secondary antibody, and nuclei were stained with Hoechst 33342.

### Lipidomics

As *Pb*Cls KO parasites grow more slowly, comparable parasitemia between WT and KO parasites at harvest was achieved by inoculating mice infected with KO parasites earlier than those infected with WT parasites. Parasite stages (mean±SD %) were ~66 ± 5% rings, 33 ± 4% trophozoites and 1 ± 0.6% schizonts in WT-GFP and ~69 ± 3% rings, 30 ± 4% trophozoites, 0.8 ± 0.6% schizonts in the *Pb*Cls KO biological replicates. Parasites were released by saponin lysis, washed and processed for lipid extraction.

### Total lipid extraction

Total lipid extractions were done using methods as previously described [97,98]. Briefly, cell pellets resuspended in 1 mL Dulbecco’s Phosphate Buffered Saline (DPBS) were made up to a 4 mL mixture of 2:1:1 choloroform (CHCl_3_): methanol (MeOH): DPBS in glass vials [containing 1 nmol of C15:0 monoacylglycerol (C15:0 MAG) and 0.5 nmol of C15:0 fatty acid (C15:0 FA) for semi-quantitative analysis of lipids]. This homogenate mixture was vortexed vigorously and centrifuged at 2500 × *g* for 15 min to separate the mixture into an organic and an aqueous phase (top) separated by a protein disk. The organic phase (~2 ml) was removed by pipetting and transferred to a new glass vial, while 50 μl of formic acid (MS grade, Fluka-Honeywell, USA) was added to the aqueous phase. This mixture was vigorously vortexed, and 2 ml of CHCl_3_ was added, followed again by vortexing. This mixture was centrifuged at 2500 × g for 15 min; the organic layer from this extraction and the previously obtained organic layer were pooled and dried under a stream of nitrogen gas. The dried lipid extracts were re-solubilized in 200 μL of 2:1 CHCl_3_: MeOH, and 10 µl of this was used for subsequent LC-MS analysis.

### Cardiolipin measurements

Cardiolipin measurement was carried out using multiple reaction monitoring transition-based analysis in an Agilent Technologies 6470 Triple Quadrupole LC/MS. Reverse-phase LC separation was achieved using a Gemini 5U C18 column (Phenomenex, 5 μm, 50 × 4.6 mm, 110 Å) coupled to a Gemini guard column (Phenomenex, 4 × 3 mm). The solvents used for LC separation were buffer A: 95:5 H_2_O/MeOH + 0.1% ammonium hydroxide and buffer B: 60:35:5 iPrOH/MeOH/H_2_O + 0.1% ammonium hydroxide. LC-MS runs were 30 min post injection, with the following gradient elution parameters: 0.3 ml/min 0% buffer B for 4 min; 0.5 mL/min linear gradient to 100% buffer B from 4-18 min; 0.5 ml/min 100% buffer B from 18-25 min; 0.5 ml/min 100-0% buffer B from 25-25.1 min; and 0.5 mL/min 0% buffer B from 25.1-30 min (re-equilibration). All LC-MS analysis was performed in negative ion mode using ESI source with the following parameters: drying and sheath gas temperature = 350°C and 375°C; drying and sheath gas flow = 11 L/min; nebulizer pressure = 45 psi; capillary voltage = 4000 V; and nozzle voltage = 1000 V. A list of all CL species targeted in this MRM method, describing the precursor parent-ion mass, the product ion targeted, other species-specific voltages and parameters, and the internal standard used can be found in S4 Table. All CL species were quantified by their respective areas under the curve, normalizing it to the area under the curve of the internal standard (C15:0 FA), and then normalizing to cell number.

### Untargeted lipid measurements

Untargeted LC-MS runs were performed using the AutoMS/MS acquisition method on an Agilent 6545 Q-TOF mass spectrometer fitted with an Agilent 1290 Infinity II UHPLC system as previously described [99]. Briefly, reverse-phase LC separation was achieved using a Gemini 5U C18 column (Phenomenex, 5 μm, 50 × 4.6 mm, 110 Å) coupled to a Gemini guard column (Phenomenex, 4 × 3 mm). The solvents used for LC separation in positive ionization mode were buffer A: 95:5 H_2_O/MeOH + 0.1% HCOOH + 10 mM ammonium formate and buffer B: 60:35:5 iPrOH/MeOH/H_2_O + 0.1% HCOOH + 10 mM ammonium formate. The solvents used for LC separation in negative ionization mode were buffer A: 95:5 H_2_O/MeOH + 0.1% ammonium hydroxide and buffer B: 60:35:5 iPrOH/MeOH/H_2_O + 0.1% ammonium hydroxide. LC-MS runs were 60 min post injection, with the following gradient elution parameters: 0.3 ml/min 0% buffer B for 5 min; 0.5 ml/min linear gradient to 100% buffer B from 5-45 min; 0.5 ml/min 100% buffer B from 45-55 min; 0.5 ml/min 100-0% buffer B from 55-55.1 min; and 0.5 ml/min 0% buffer B from 55.1-60 min (re-equilibration). The autosampler (samples) and column temperatures were maintained 10°C and 40°C, respectively. MS acquisition was conducted using a dual Agilent jet stream electrospray ionization (AJS ESI) source in positive and negative ionization mode. MS parameters: drying gas and sheath gas temperature at 320°C, drying gas and sheath gas flow at 10 L/min, nebulizer pressure at 45 psi, capillary voltage (VCap) at 4000 V, nozzle voltage at 1000 V, and fragmentor voltage at 150 V. Precursor selections were carried out with a maximum of 4 precursor ions per cycle with fixed collision energies set at 5 eV and 15 eV.

For data analysis, a library for different classes of lipids were curated as a Personal Compound Database Library (PCDL) using MassHunter PCDL manager B.08.00 (Agilent Technologies, USA). This library was curated using the METLIN lipid library as reference. The data files were processed in Agilent MassHunter Qualitative Analysis 10.0 using the PCDL library; all the peaks were validated based on relative retention times and fragments obtained, if any. All detected species were within a mass accuracy of 15 ppm and quantified by measuring the area under the curve (AUC) for different lipid species. The values were then normalized to levels of internal standards (C15:0 MAG and C15:0 FA for positive and negative mode respectively) and the cell number for the samples.

### Determination of oxygen consumption rate (OCR)

OCR measurement was performed as described [100,101] using Agilent Seahorse XFe24 Analyzer and Agilent XF Cell Mito Stress Kit with some modifications. A sensor cartridge was hydrated at 37°C overnight prior to the experiment. Wild type and *Pb*Cls KO parasites primarily at trophozoite stage were harvested with pre-warmed 0.05% saponin and then washed twice with pre-warmed 1XPBS. The cells were resuspended in 1X mitochondrial assay solution (MAS: 220 mM mannitol, 2 mM HEPES, 10 mM malate, 70 mM sucrose, 5 mM MgCl_2_,10 mM KH_2_PO_4_, 1 mM EGTA, 0.2% [wt/vol] fatty acid-free BSA) and cell numbers were adjusted to 5 × 10^7^ cells/ml. Cells were permeabilized with 0.002% (w/v) digitonin (Sigma Aldrich, USA) and 100 µl of this suspension was seeded per well in XFe24 microplate pre-coated with Cell-Tak (Corning, USA). The plate was centrifuged at 800 × g for 10 min. Additional 75 µl of 1XMAS was added without disturbing attached parasites. Inhibitors were prepared in 1XMAS buffer. After placing the sensor cartridge on utility plate, injection ports were filled with 25 µl of inhibitors (Port A: FCCP and Port B: Antimycin at final concentration of 4 µM and 0.5 µM, respectively). The measurement of OCR included 5 cycles of 20 s mixing, 1 min waiting, and 2.5 min for both basal respiration and measurement after injection of inhibitors.

### Statistical analyses

All statistical analyses were performed using GraphPad Prism 9 Software. Data were analyzed using unpaired two-tailed Student’s t-test, multiple t-test, one-way ANOVA or Mann-Whitney non-parametric two-tailed test. Statistical significance is indicated as: P < 0.05 (*), P < 0.01 (**), P < 0.001 (***), P < 0.0001 (****); n.s., not significant.

## Supporting information

S1 Fig Sequence alignment and recombinant protein. (**A**) ClustalW alignment (https://prosite.expasy.org) of *Pf*Cls and its homologs from *Plasmodium berghei*, *Cryptosporidium parvum*, *Trypanosoma brucei*, *Escherichia coli* (ClsB) and *Streptococcus pneumoniae.* Green and blue arrows indicate the first and last residues of recombinant *Pf*Cls and *Pf*Cls-CTD, respectively. The phospholipase D (PLD) domains with the conserved HKD (*) motifs (H-x-K-x_4_-D-x_6_-G-S-x-N) are indicated by a blue line. (**B**) Prediction of *Pf*Cls structure from AlphaFold (https://alphafold.ebi.ac.uk/). Regions with per-residue model confidence score (pLDDT) below 50 may be unstructured in isolation. (**C**) Coomassie-stained SDS-PA gel of purified *Pf*Cls-CTD (i) and its western blot with anti-6XHis Ab (ii). (**D**) Western blots of equal quantity of lysates from *E. coli* cells expressing *Pf*Cls-CTD and those transformed with the vector alone (EV). Blots were probed with anti- *Pf*Cls-CTD serum (I) or pre-immune serum (PI).
(PDF)

S2 Fig Membrane association of *Pf*Cls. (**A**) TMHMM (https://services.healthtech.dtu.dk/services/TMHMM-2.0/) prediction of transmembrane domain in *Pf*Cls. No transmembrane helices are detected, although a hydrophobic region is identified between aa 163–185. (**B**) Detection of recombinant *Pf*Cls in induced *E. coli* cells transformed with empty vector pET23a (EV) or pET23a-*Pf*Cls (*Pf*Cls) by IFA using anti-6XHis Ab. *Pf*Cls at bacterial cell poles and septum is indicated by white arrows. (**C**) Differential protein extraction from *P. falciparum* trophozoites using Tris and carbonate buffers and TritonX-100 to determine *Pf*Cls partitioning in cytosolic, extrinsic or intrinsic membrane protein fractions, respectively. β-actin served as control protein. Blots were probed with anti-*Pf*Cls and anti-β-actin Abs. (**D**) Crosslinking of parasite proteins using DSP followed by release of complex constituents by DTT, and detection by western blotting using anti-*Pf*Cls Ab. The arrow indicates a large complex near the well in the cross-linked sample without DTT treatment (lane 1). (**E**) Thermolysin protection assay for subcellular partitioning of *Pf*Cls. Western blots of lysates from parasites treated with digitonin and digested with thermolysin in the presence or absence of EDTA were probed with anti-*Pf*Cls antisera, anti-*Pf*HU antisera and anti-α-tubulin antibody.
(PDF)

S3 Fig Biochemical activity of *Pf*Cls. (**A**) Western blot of lysate of *E. coli* cells expressing recombinant *Pf*Cls probed with anti-6XHis Ab detects the full-length expressed protein (~66 kDa) and lower degradation products. (**B**) Size exclusion chromatography profile (S75 column) of affinity-purified recombinant *Pf*Cls. The full-length protein eluted as peak 1, with peak 2 primarily comprising degradation products. Inset, Coomassie-stained SDS-PAGE of peak 1 fraction. The corresponding chromatography plot for standard protein molecular weight markers is shown. (**C**) CD spectrum of purified *Pf*Cls. (**D**) Control blot for protein-lipid overlay assay in Fig 3B. Blots with spotted phospholipids were incubated with 6XHis-tagged *Pf*Exo and probed with anti-6XHis Ab. (**E**) TLC of positive control reactions with *P. syringae* Cls (1 µM) which catalyzes the formation of CL from PG + PE. The first three lanes contain PG, PE, and CL as standards. (**F**) Assay of *Pf*Cls activity under identical reaction conditions as the *Ps*Cls control (E), except that 5 µM *Pf*Cls was used in the reactions. CL formation was not seen with PG alone or with PG + PE. Representative images from three repeat experiments are shown for (E) and (F). (**G**) TLC to check PE formation from CDP-DAG + EA using purified *Ps*Cls and *Pf*Cls in the presence or absence of 2 mM ATP. Only PA formation, generated from hydrolysis of CDP-DAG (PLD-like activity), is seen.
(PDF)

S4 Fig Generation of *Pb*Cls knockout and complemented parasite lines. (**A**) Schematic representation of the strategy used to disrupt the *Pb*Cls gene by double-crossover homologous recombination. Two homologous fragments (F1 and F2) were cloned into the pBC-GFP-hDHFR:yFCU vector. Arrows and lollipops denote the 5’ and 3’ UTRs, respectively. (**B**) Fluorescence microscopy showing GFP expression in blood-stage *Pb*Cls KO parasites. (**C**) PCR analysis confirming correct site-specific integration and the absence of the WT *Pb*Cls ORF in KO lines. Primer pair 2183/1225 was used to amplify the 5’ integration, primer pair 1215/2184 for the 3’ integration, and primer pair 2206/2207 for amplification of the endogenous locus. (**D**) Schematic of the genetic complementation approach used to restore *Pb*Cls locus. (**E**) PCR amplification confirming the presence of the *Pb*Cls ORF in complemented parasite lines.
(PDF)

S5 Fig *Pb*Cls expression during blood and liver stages. (**A**) Western blot with anti-*Pf*Cls serum detects *Pb*Cls in WT-GFP but not in *Pb*Cls KO. *Pb*Hsp70, detected by anti-*Pb*Hsp70 Ab, was used as loading control. (**B** and **C**) *Pb*Cls expression in blood (B) and liver (C) stages in WT and *Pb*Cls KO parasites. (**D**) PCR at different stages of *Pb*Cls KO parasites to confirm the absence of the WT *Pb*Cls ORF. MG Oocyst, mid-gut oocysts; SG Spz, salivary gland sporozoites; PP, patent parasites from blood; NTC, no-template control.
(PDF)

S6 Fig Lipidomic analysis and OCR of *Pb*Cls KO and WT. (**A-E**) Total PG, PA, PE, PC and PS levels in *Pb*Cls KO compared to WT-GFP parasites. Mean ± SD of three independent biological replicates are plotted. ns, not significant. P-values are indicated by *. (**F**) Levels of PG and PA molecular species in WT-GFP and *Pb*Cls KO parasites. (**G**) Levels of PE molecular species in WT-GFP and *Pb*Cls KO parasites. (**H**) Levels of PC molecular species in WT-GFP and *Pb*Cls KO parasites. (**I**) Levels of PS molecular species in WT-GFP and *Pb*Cls KO parasites. Multiple t-test (with 5% Benjamini-Krieger-Yekutieli FDR correction) was applied in (F) to (I). (**J**) Oxygen consumption rate of WT-GFP and *Pb*Cls KO parasites over time with sequential addition of FCCP and Antimycin. Mean ± SD of the OCR measurement of parasites taken from three mice per group (with three technical replicates per mouse) are plotted.
(PDF)

S7 Fig Dispensability of *Pb*Cls for parasite development in mosquitoes. (**A**) Comparable ookinete development in WT-GFP and *Pb*Cls KO parasites. (**B**) Oocyst development was similar across all parasite lines. (**C**) Representative images of oocysts undergoing sporogony. (**D**) Salivary glands contained GFP-expressing sporozoites in all groups.
(PDF)

S8 Fig *Pb*Cls KO sporozoites efficiently invade hepatocytes. (**A**) To assess hepatocyte invasion, HepG2 cells infected with WT-GFP or *Pb*Cls KO sporozoites were stained with anti-CSP antibody before and after permeabilization at 1.5 hpi. Non-invaded extracellular sporozoites were detected by staining prior to permeabilization (red), whereas all sporozoites were labeled after permeabilization (green). (**B**) Quantification revealed no significant difference in invasion efficiency between WT GFP and *Pb*Cls KO sporozoites (P = 0.1459). Data represent mean ± SEM from two independent experiments. Statistical significance was determined using one-way ANOVA.
(PDF)

S9 Fig Schematic of reactions catalyzed by *Pf*Cls and characterized bacterial homologs from *E. coli* (*Ec*ClsA/B/C) and *P. syringae* (*Ps*Cls). ‘?’ indicates reactions catalyzed by one or more of the *Ec*Cls isoenzymes and/or *Ps*Cls but not by recombinant *Pf*Cls used in this study.
(PDF)

S1 Table Peptide mass spectrometry of recombinant *Pf*Cls. (XLSX)

S2 Table Phylogeny sequences. (XLSX)

S3 Table PCR primers. (XLSX)

S4 Table Multiple Reaction Monitoring Table and source data for lipidomic analysis in Figs 4 and S6. (XLSX)
