# Supplementary figures and images for: A bacterial-type cardiolipin synthase in Plasmodium spp. supports mitochondrial respiration and is important for liver stage maturation
Source: PLoS Pathog. 2026 May 11;22(5):e1014215. doi: 10.1371/journal.ppat.1014215 (PMC13175493; doi:10.1371/journal.ppat.1014215)

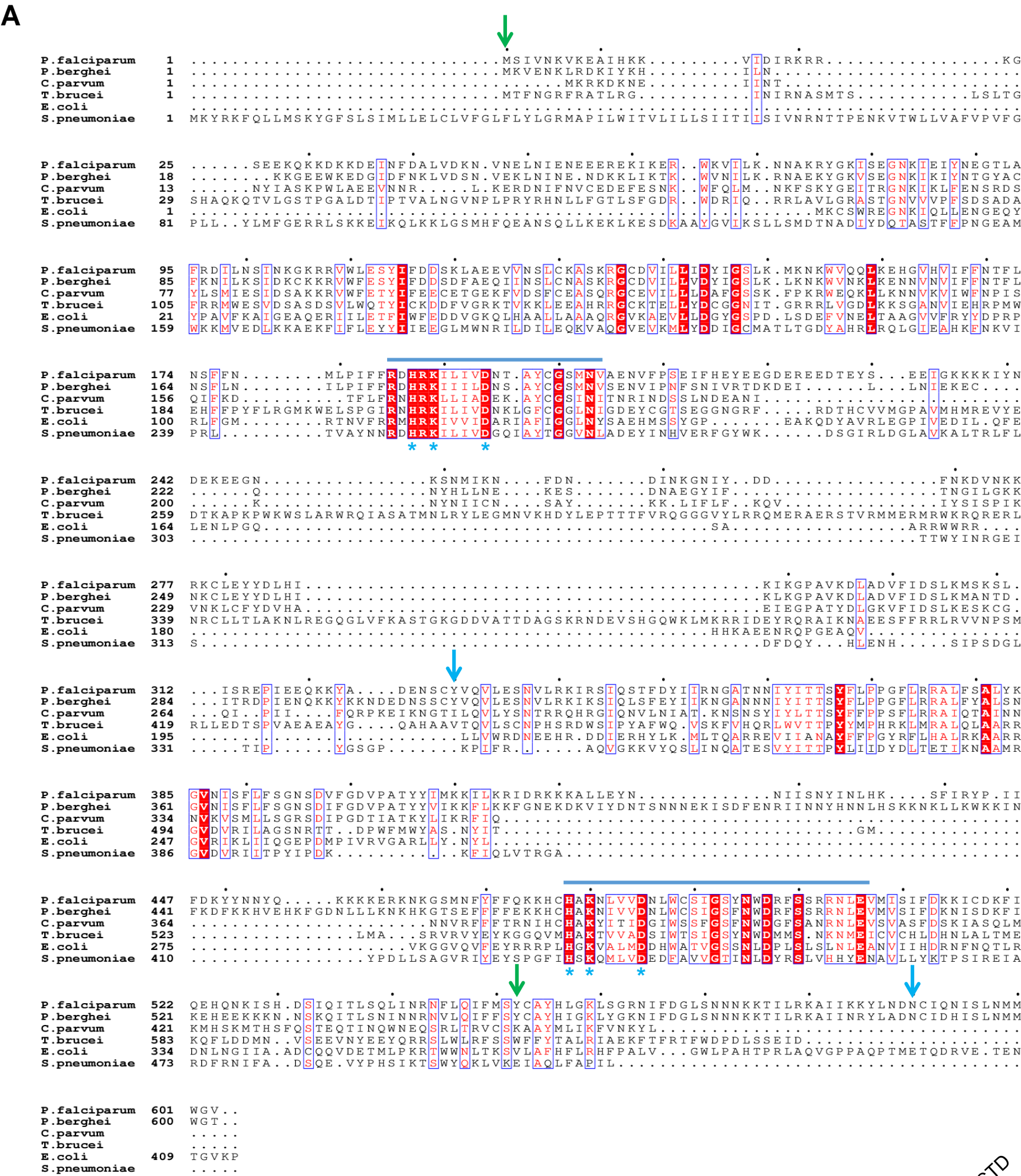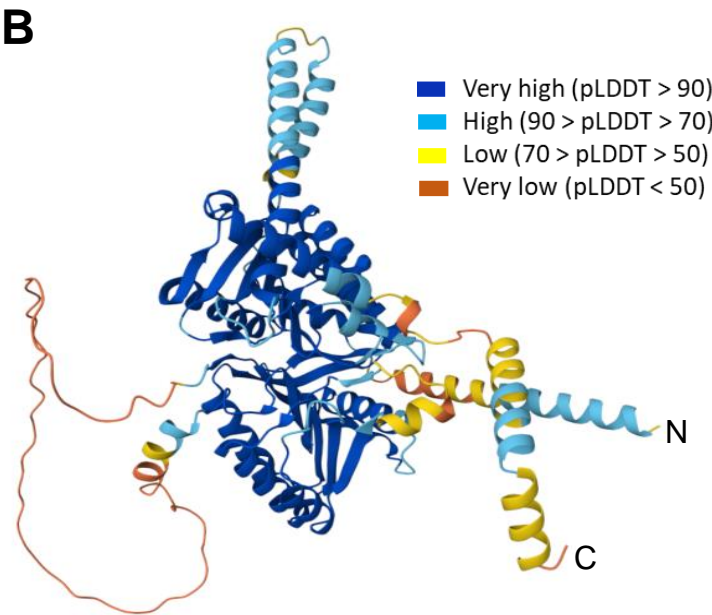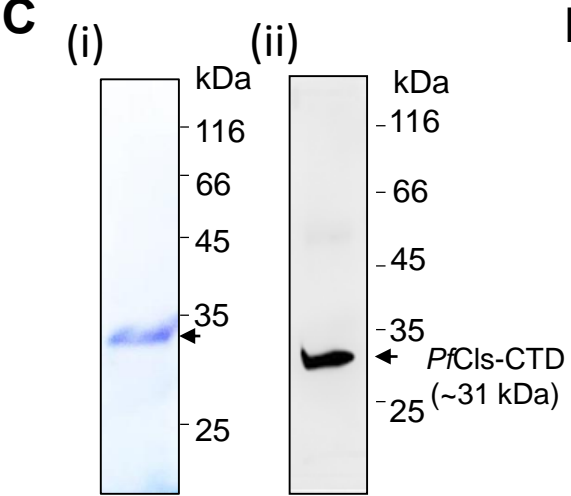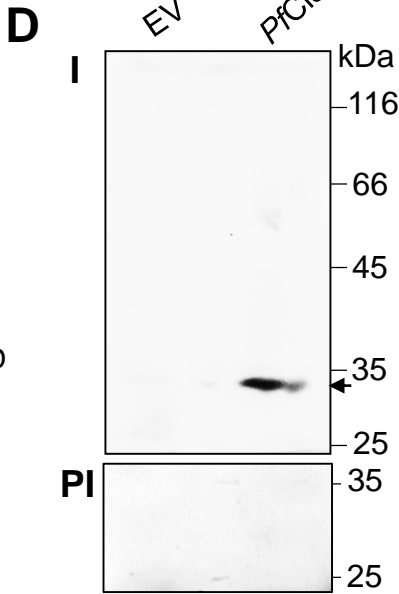

Supplement: S1 Fig — (A) ClustalW alignment (https://prosite.expasy.org) of PfCls and its homologs from Plasmodium berghei, Cryptosporidium parvum, Trypanosoma brucei, Escherichia coli (ClsB) and Streptococcus pneumoniae. Green and blue arrows indicate the first and last residues of recombinant PfCls and PfCls-CTD, respectively. The phospholipase D (PLD) domains with the conserved HKD (*) motifs (H-x-K-x4-D-x6-G-S-x-N) are indicated by a blue line. (B) Prediction of PfCls structure from AlphaFold (https://alphafold.ebi.ac.uk/). Regions with per-residue model confidence score (pLDDT) below 50 may be unstructured in isolation. (C) Coomassie-stained SDS-PA gel of purified PfCls-CTD (i) and its western blot with anti-6XHis Ab (ii). (D) Western blots of equal quantity of lysates from E. coli cells expressing PfCls-CTD and those transformed with the vector alone (EV). Blots were probed with anti- PfCls-CTD serum (I) or pre-immune serum (PI). (PDF) [file ppat.1014215.s001.pdf]

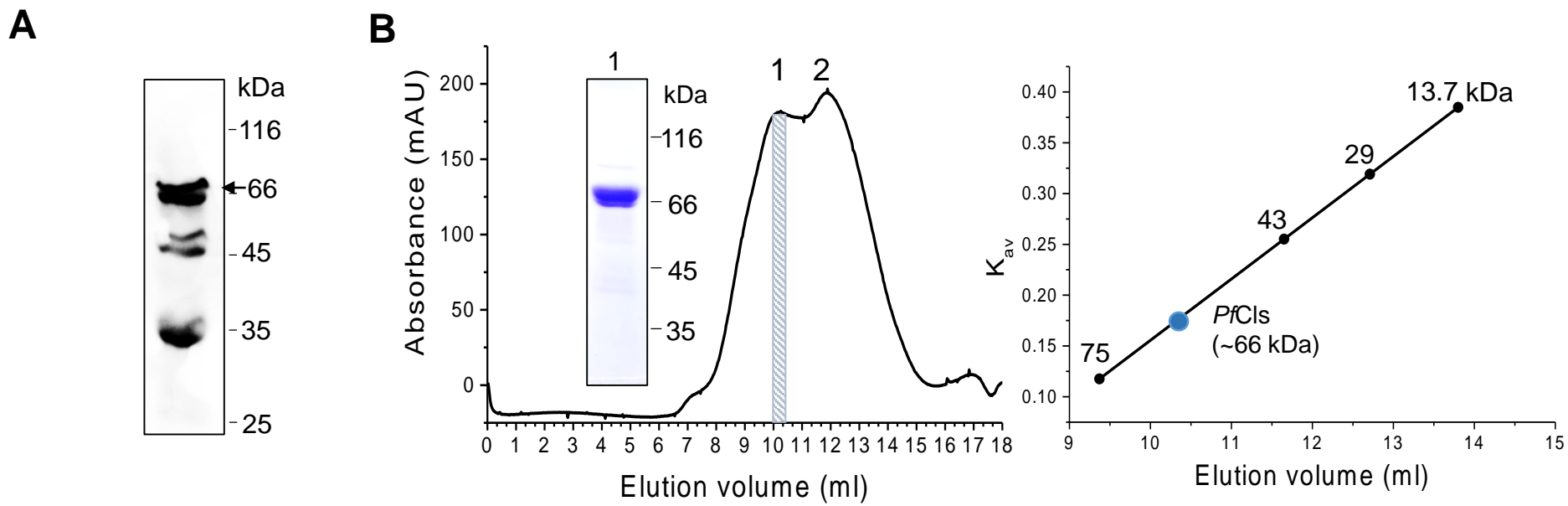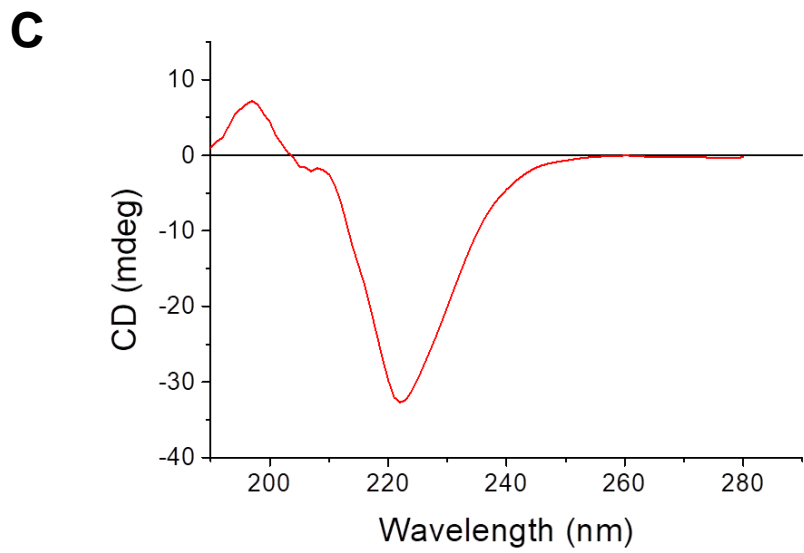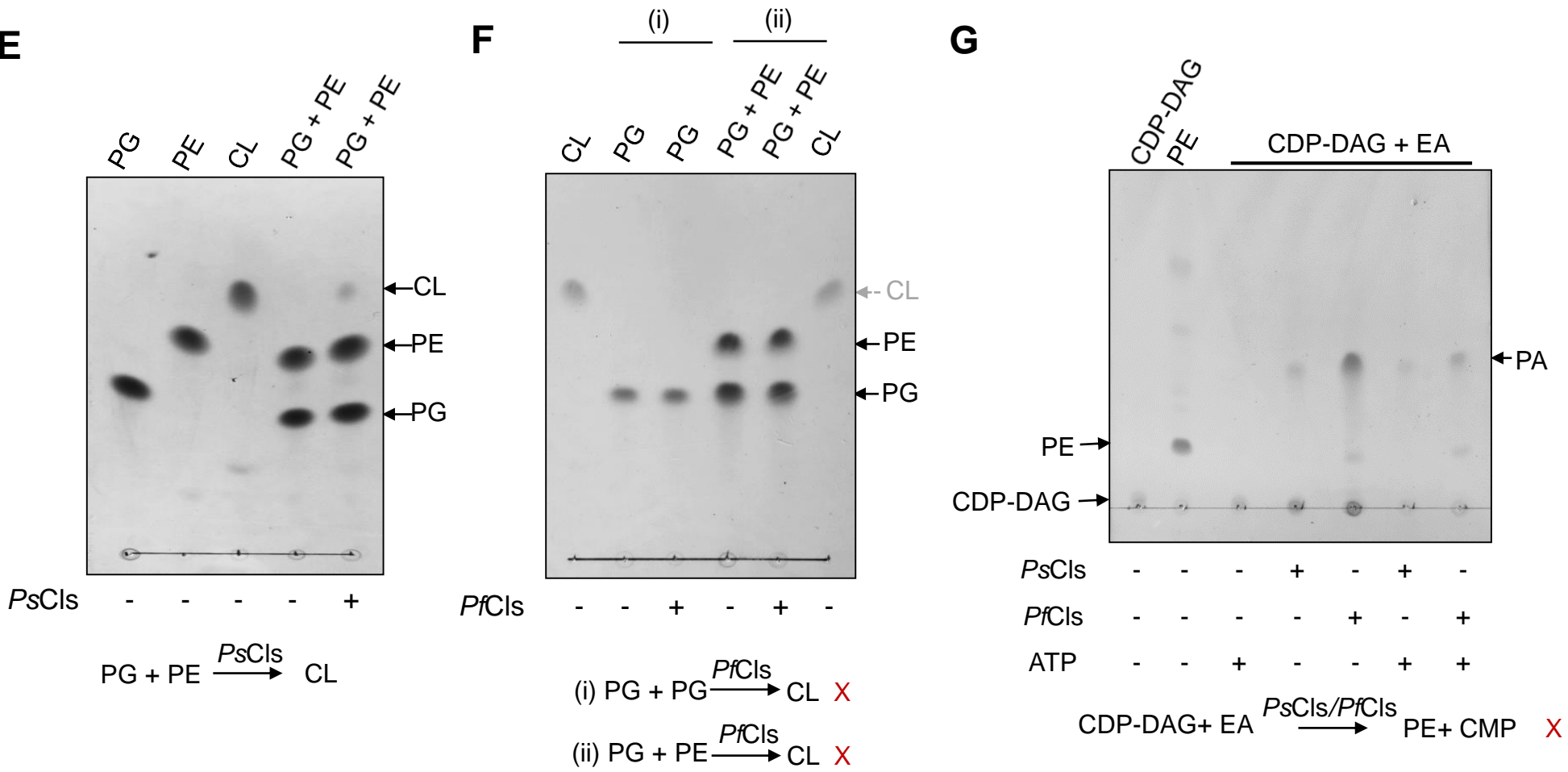

Supplement: S3 Fig — (A) Western blot of lysate of E. coli cells expressing recombinant PfCls probed with anti-6XHis Ab detects the full-length expressed protein (~66 kDa) and lower degradation products. (B) Size exclusion chromatography profile (S75 column) of affinity-purified recombinant PfCls. The full-length protein eluted as peak 1, with peak 2 primarily comprising degradation products. Inset, Coomassie-stained SDS-PAGE of peak 1 fraction. The corresponding chromatography plot for standard protein molecular weight markers is shown. (C) CD spectrum of purified PfCls. (D) Control blot for protein-lipid overlay assay in Fig 3B. Blots with spotted phospholipids were incubated with 6XHis-tagged PfExo and probed with anti-6XHis Ab. (E) TLC of positive control reactions with P. syringae Cls (1 µM) which catalyzes the formation of CL from PG + PE. The first three lanes contain PG, PE, and CL as standards. (F) Assay of PfCls activity under identical reaction conditions as the PsCls control (E), except that 5 µM PfCls was used in the reactions. CL formation was not seen with PG alone or with PG + PE. Representative images from three repeat experiments are shown for (E) and (F). (G) TLC to check PE formation from CDP-DAG + EA using purified PsCls and PfCls in the presence or absence of 2 mM ATP. Only PA formation, generated from hydrolysis of CDP-DAG (PLD-like activity), is seen. (PDF) [file ppat.1014215.s003.pdf]

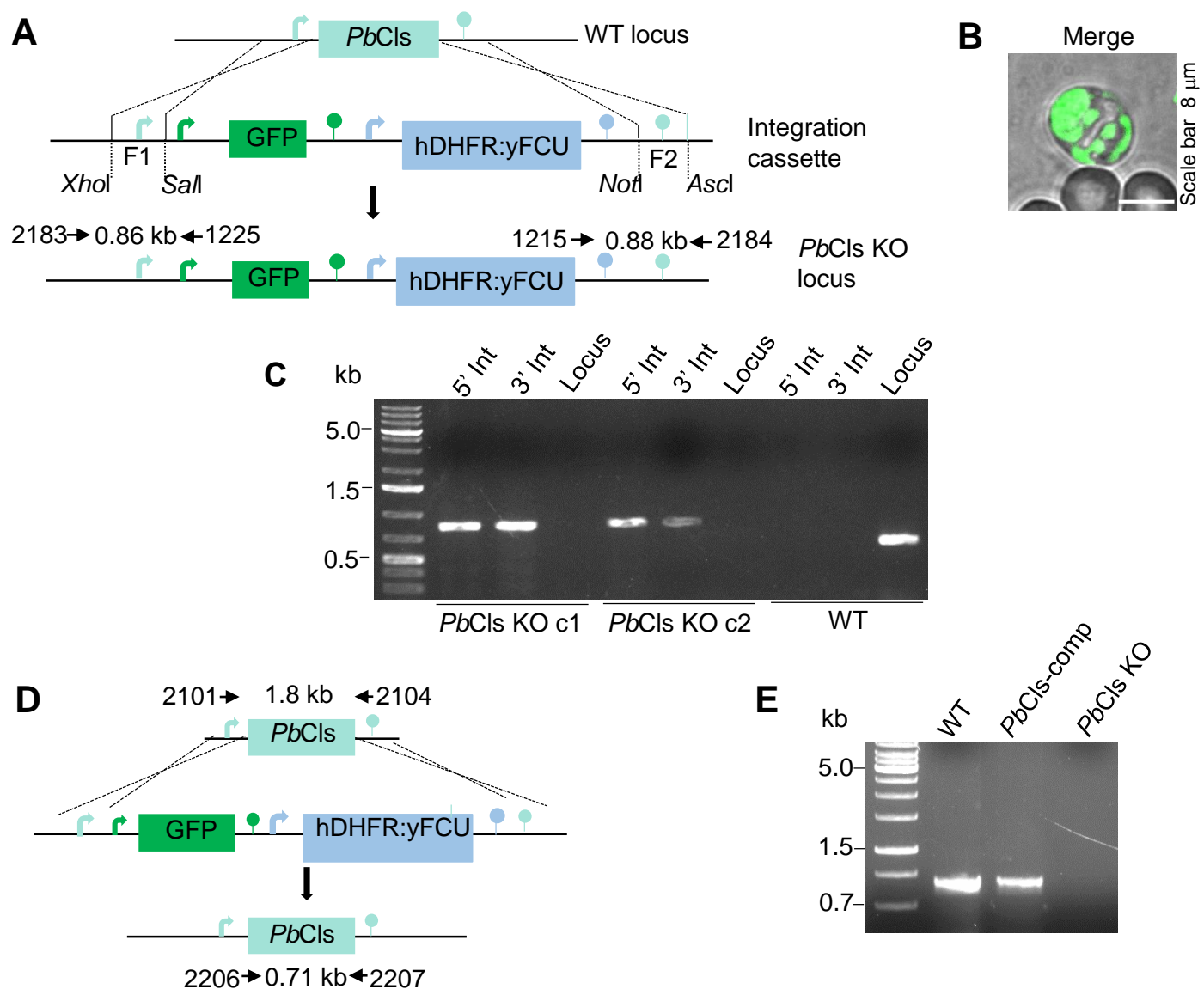

Supplement: S4 Fig — (A) Schematic representation of the strategy used to disrupt the PbCls gene by double-crossover homologous recombination. Two homologous fragments (F1 and F2) were cloned into the pBC-GFP-hDHFR:yFCU vector. Arrows and lollipops denote the 5’ and 3’ UTRs, respectively. (B) Fluorescence microscopy showing GFP expression in blood-stage PbCls KO parasites. (C) PCR analysis confirming correct site-specific integration and the absence of the WT PbCls ORF in KO lines. Primer pair 2183/1225 was used to amplify the 5’ integration, primer pair 1215/2184 for the 3’ integration, and primer pair 2206/2207 for amplification of the endogenous locus. (D) Schematic of the genetic complementation approach used to restore PbCls locus. (E) PCR amplification confirming the presence of the PbCls ORF in complemented parasite lines. (PDF) [file ppat.1014215.s004.pdf]

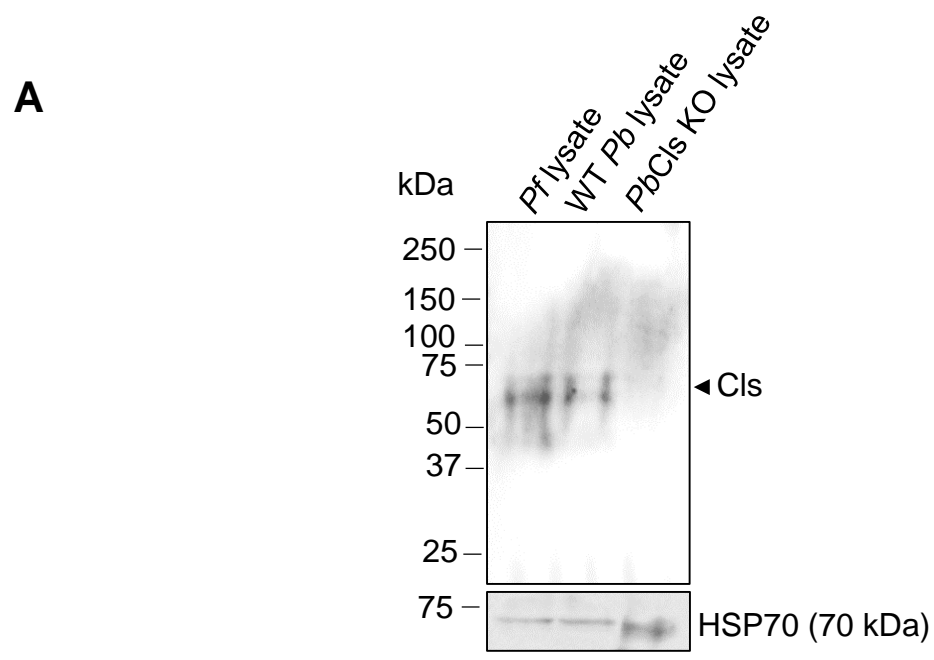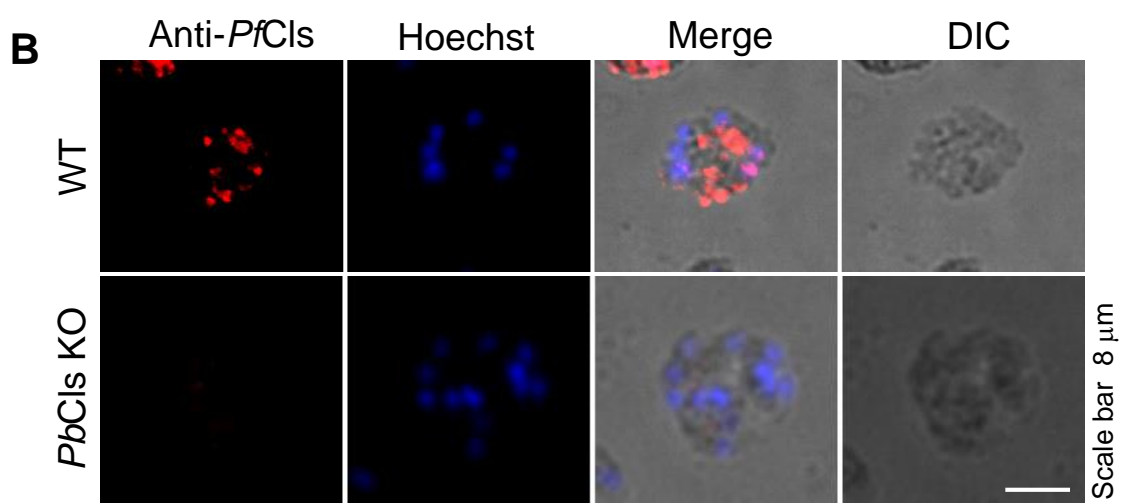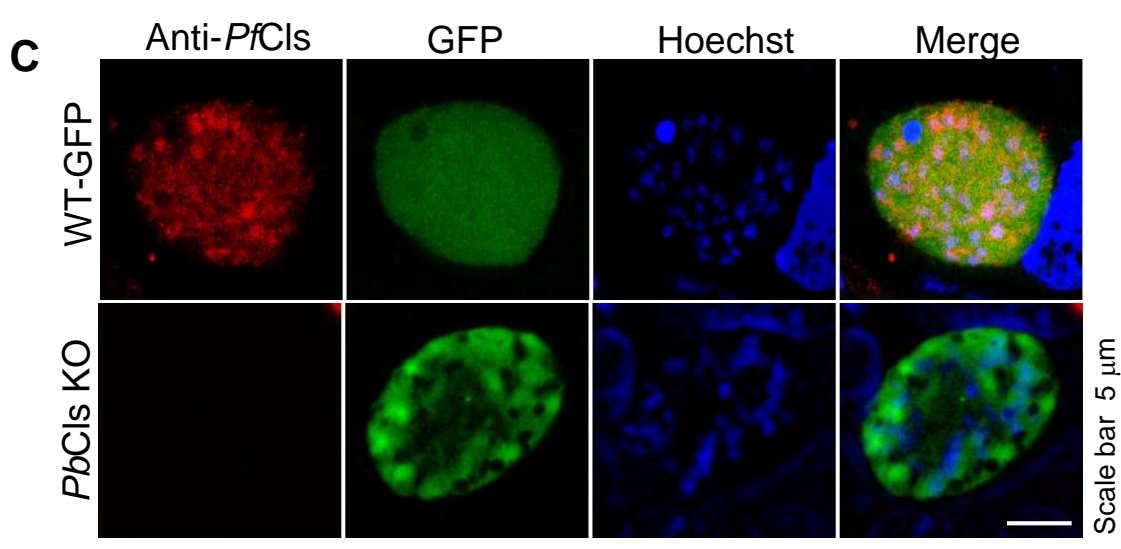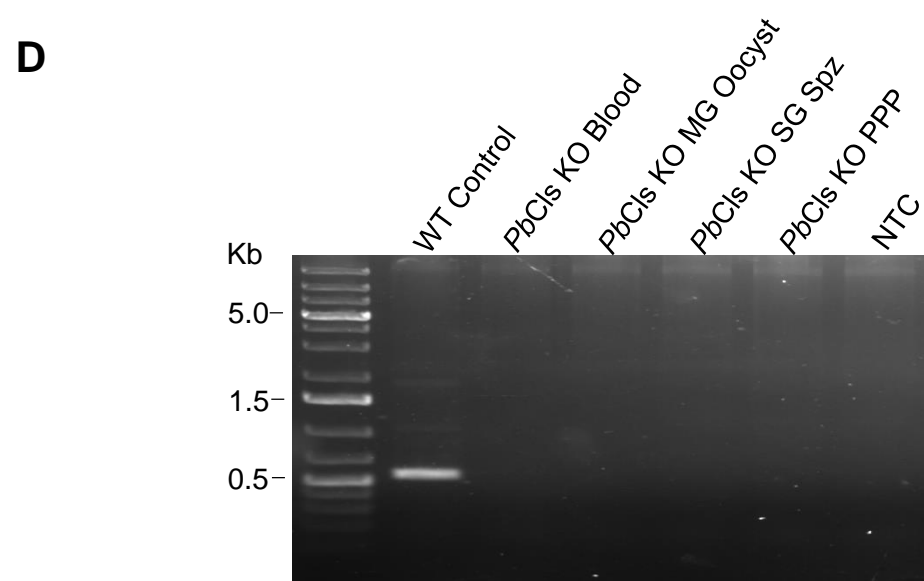

Supplement: S5 Fig — (A) Western blot with anti-PfCls serum detects PbCls in WT-GFP but not in PbCls KO. PbHsp70, detected by anti-PbHsp70 Ab, was used as loading control. (B and C) PbCls expression in blood (B) and liver (C) stages in WT and PbCls KO parasites. (D) PCR at different stages of PbCls KO parasites to confirm the absence of the WT PbCls ORF. MG Oocyst, mid-gut oocysts; SG Spz, salivary gland sporozoites; PP, patent parasites from blood; NTC, no-template control. (PDF) [file ppat.1014215.s005.pdf]

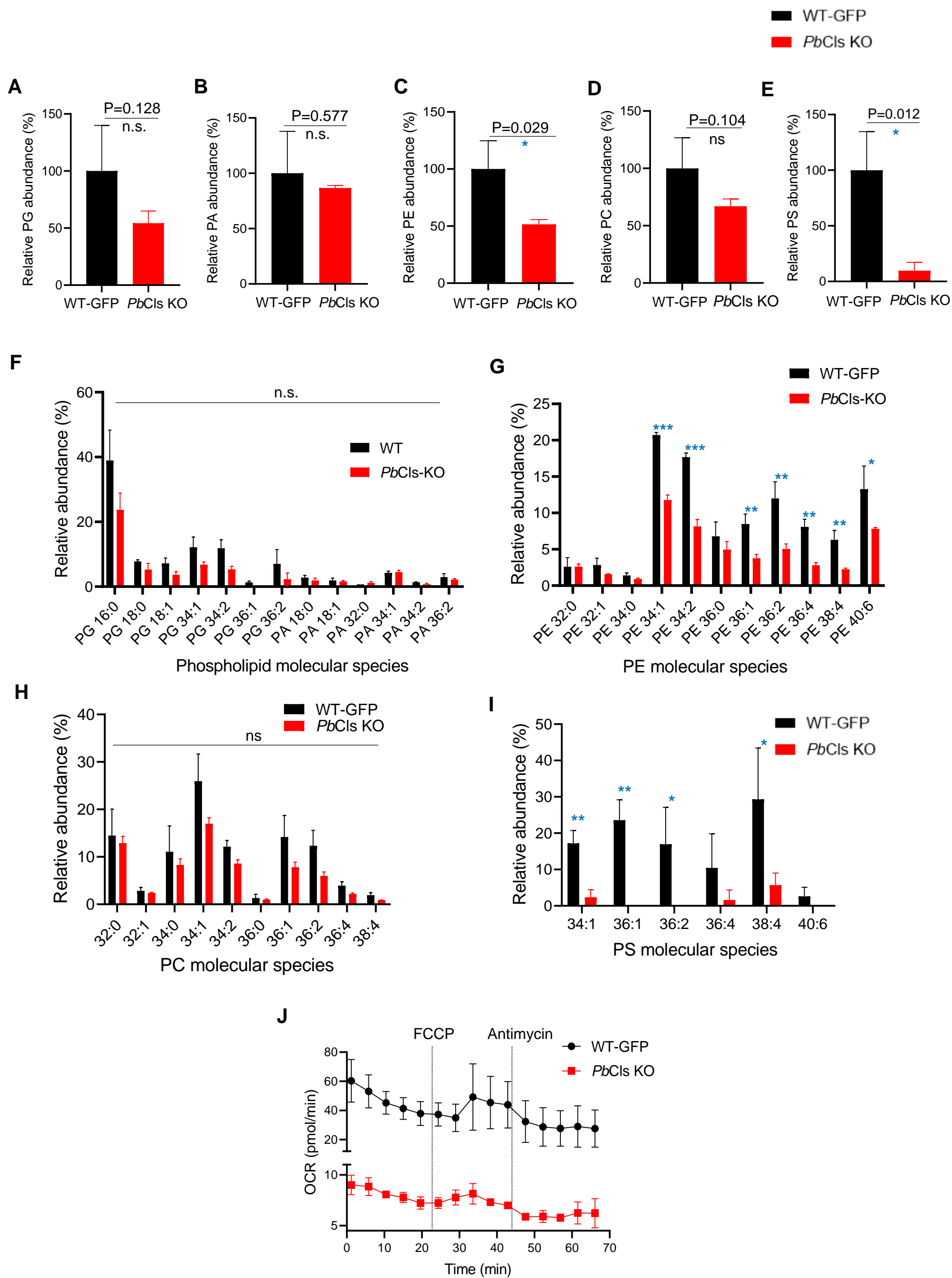

Supplement: S6 Fig — (A-E) Total PG, PA, PE, PC and PS levels in PbCls KO compared to WT-GFP parasites. Mean ± SD of three independent biological replicates are plotted. ns, not significant. P-values are indicated by *. (F) Levels of PG and PA molecular species in WT-GFP and PbCls KO parasites. (G) Levels of PE molecular species in WT-GFP and PbCls KO parasites. (H) Levels of PC molecular species in WT-GFP and PbCls KO parasites. (I) Levels of PS molecular species in WT-GFP and PbCls KO parasites. Multiple t-test (with 5% Benjamini-Krieger-Yekutieli FDR correction) was applied in (F) to (I). (J) Oxygen consumption rate of WT-GFP and PbCls KO parasites over time with sequential addition of FCCP and Antimycin. Mean ± SD of the OCR measurement of parasites taken from three mice per group (with three technical replicates per mouse) are plotted. (PDF) [file ppat.1014215.s006.pdf]

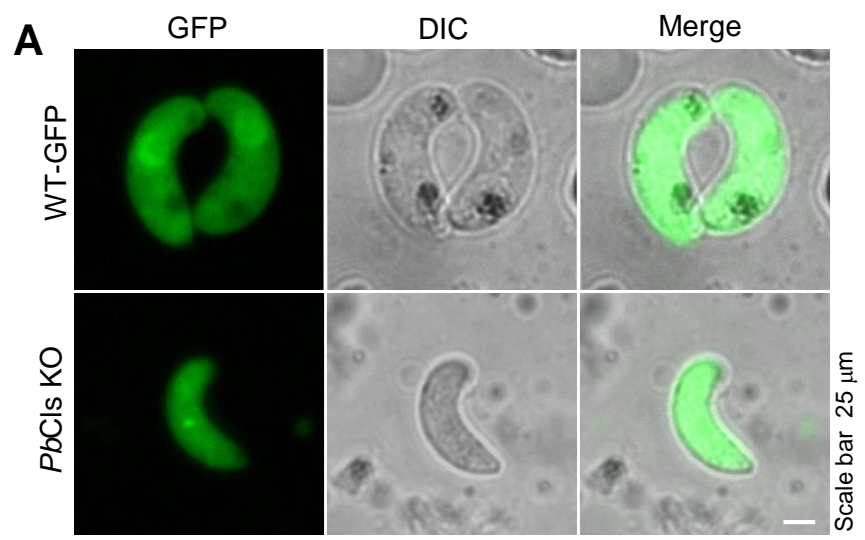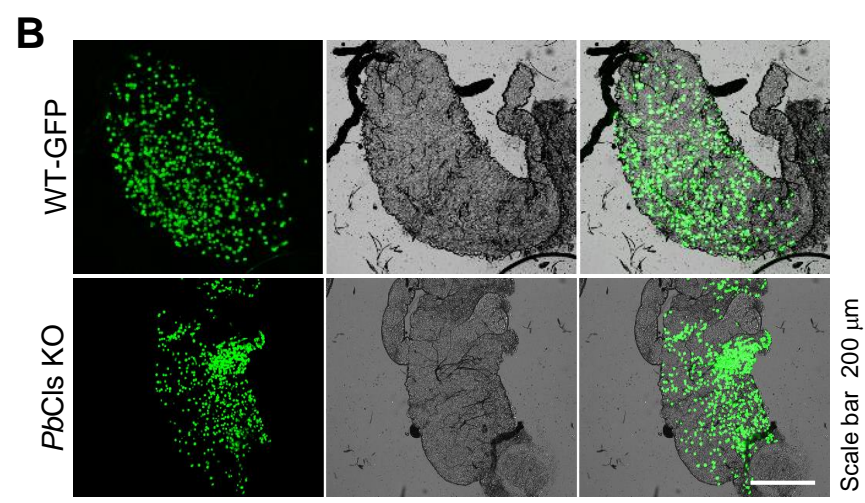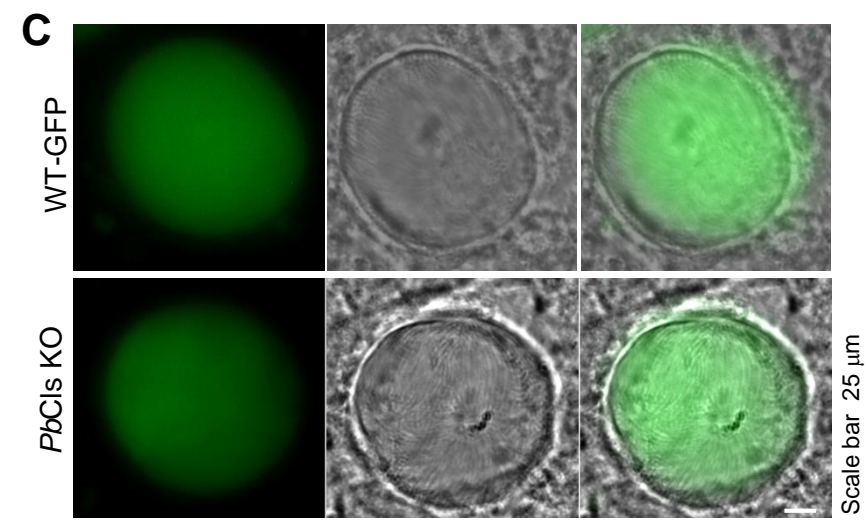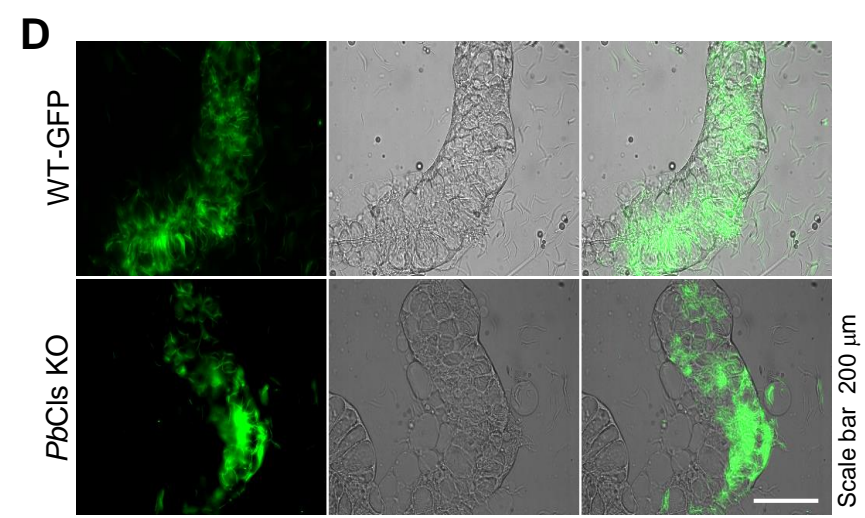

Supplement: S7 Fig — (A) Comparable ookinete development in WT-GFP and PbCls KO parasites. (B) Oocyst development was similar across all parasite lines. (C) Representative images of oocysts undergoing sporogony. (D) Salivary glands contained GFP-expressing sporozoites in all groups. (PDF) [file ppat.1014215.s007.pdf]

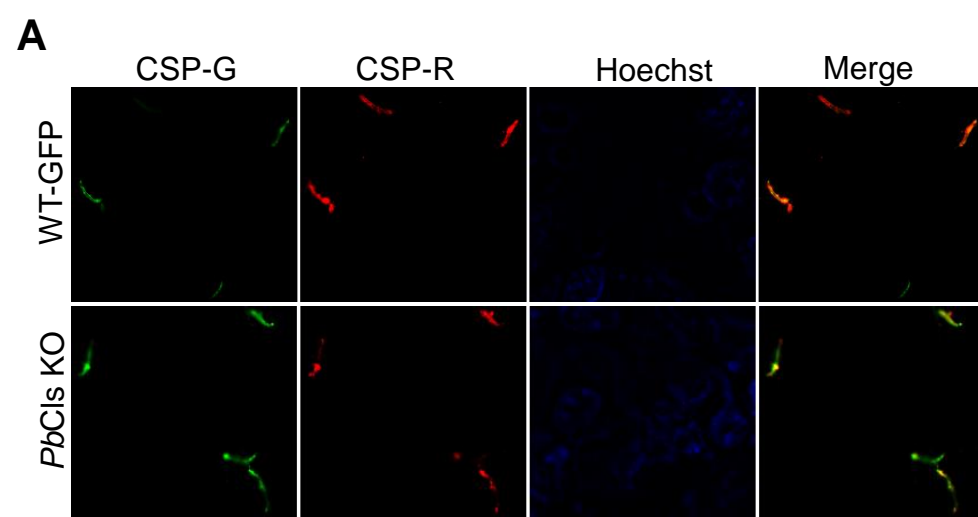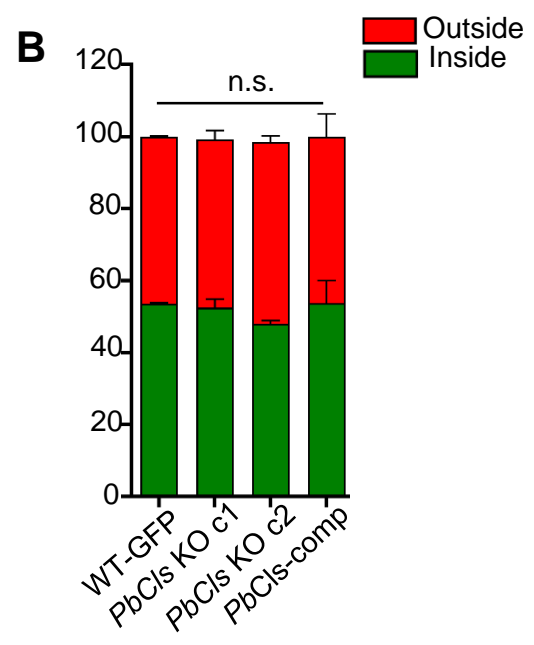

Supplement: S8 Fig — (A) To assess hepatocyte invasion, HepG2 cells infected with WT-GFP or PbCls KO sporozoites were stained with anti-CSP antibody before and after permeabilization at 1.5 hpi. Non-invaded extracellular sporozoites were detected by staining prior to permeabilization (red), whereas all sporozoites were labeled after permeabilization (green). (B) Quantification revealed no significant difference in invasion efficiency between WT GFP and PbCls KO sporozoites (P = 0.1459). Data represent mean ± SEM from two independent experiments. Statistical significance was determined using one-way ANOVA. (PDF) [file ppat.1014215.s008.pdf]

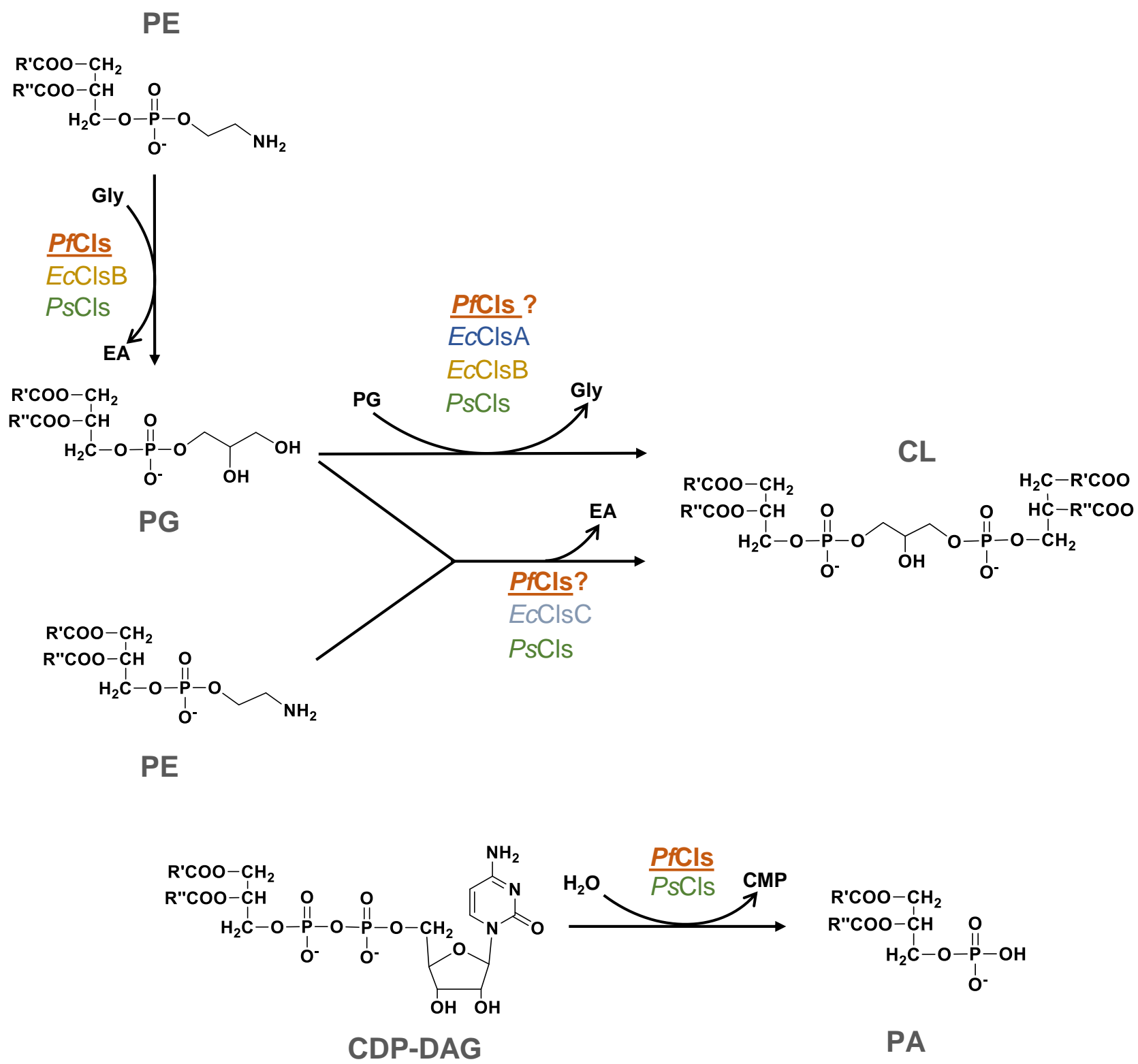

Supplement: S9 Fig — ‘?’ indicates reactions catalyzed by one or more of the EcCls isoenzymes and/or PsCls but not by recombinant PfCls used in this study. (PDF) [file ppat.1014215.s009.pdf]
