# Supplementary material for: A bacterial-type cardiolipin synthase in Plasmodium spp. supports mitochondrial respiration and is important for liver stage maturation
Source: PLoS Pathog. 2026 May 11;22(5):e1014215. doi: 10.1371/journal.ppat.1014215 (PMC13175493; doi:10.1371/journal.ppat.1014215)
Supplement: S2 Fig — (A) TMHMM (https://services.healthtech.dtu.dk/services/TMHMM-2.0/) prediction of transmembrane domain in PfCls. No transmembrane helices are detected, although a hydrophobic region is identified between aa 163–185. (B) Detection of recombinant PfCls in induced E. coli cells transformed with empty vector pET23a (EV) or pET23a-PfCls (PfCls) by IFA using anti-6XHis Ab. PfCls at bacterial cell poles and septum is indicated by white arrows. (C) Differential protein extraction from P. falciparum trophozoites using Tris and carbonate buffers and TritonX-100 to determine PfCls partitioning in cytosolic, extrinsic or intrinsic membrane protein fractions, respectively. β-actin served as control protein. Blots were probed with anti-PfCls and anti-β-actin Abs. (D) Crosslinking of parasite proteins using DSP followed by release of complex constituents by DTT, and detection by western blotting using anti-PfCls Ab. The arrow indicates a large complex near the well in the cross-linked sample without DTT treatment (lane 1). (E) Thermolysin protection assay for subcellular partitioning of PfCls. Western blots of lysates from parasites treated with digitonin and digested with thermolysin in the presence or absence of EDTA were probed with anti-PfCls antisera, anti-PfHU antisera and anti-α-tubulin antibody. (PDF) [file ppat.1014215.s002.pdf]

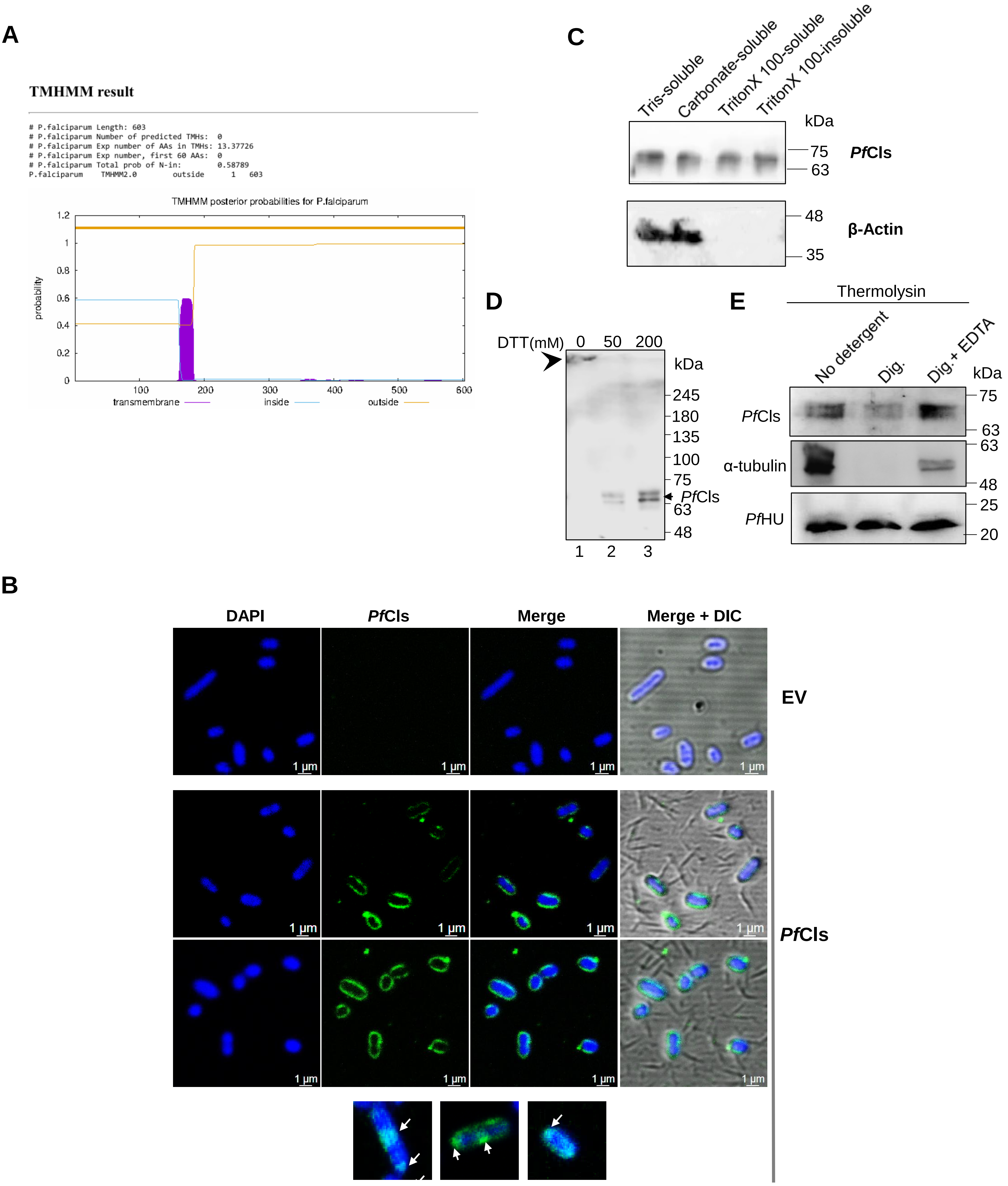

A
TMHMM result
P.falciparum Length: 603
P.falciparum Number of predicted TMHs: 0
P.falciparum Exp number of AAs in TMHs: 13.37726
P.falciparum Exp number, first 60 AAs: 0
P.falciparum Total prob of N-in: 0.58789
P.falciparum TMHMM2.0 outside 1 603
TMHMM posterior probabilities for P.falciparum
probability
transmembrane
inside
outside
C
Tris-soluble
Carbonate-soluble
TritonX 100-soluble
TritonX 100-insoluble
kDa
75
63
PfCls
48
35
β-Actin
D
DTT(mM) 0 50 200
kDa
245
180
135
100
75
PfCls
63
48
1 2 3
E
Thermolysin
No detergent
Dig.
Dig.+ EDTA
kDa
PfCls
α-tubulin
PfHU
B
DAPI
PfCls
Merge
Merge + DIC
EV
1 µm
